# Off to a good start: current gaps and priorities in early-life microbiome research

**DOI:** 10.1093/femsre/fuag010

**Published:** 2026-03-09

**Authors:** Veronika Kuchařová Pettersen, Alise Jany Ponsero, Ching Jian, Alona Riumin, Alexander Kurilshikov, Sabrina John Moyo, Museveni Justine, Claus Klingenberg, Justine Debelius, Mireia Valles-Colomer, Hanna Theodora Noordzij, Alexandra Zhernakova, Katri Korpela, Maria Esteban-Torres, Nele Brusselaers

**Affiliations:** Host-Microbe Interactions Research Group, Department of Medical Biology, Faculty of Health Sciences, UiT The Arctic University of Norway, 9037 Tromsø, Norway; Research Group for Child and Adolescent Health, Department of Clinical Medicine, Faculty of Health Sciences, UiT The Arctic University of Norway, 9037 Tromsø, Norway; Department of Bacteriology and Immunology, Faculty of Medicine, University of Helsinki, 00014 Helsinki, Finland; Core Bioinformatics, Quadram Institute of Biosciences, Norwich NR4 7UQ, United Kingdom; Department of Bacteriology and Immunology, Faculty of Medicine, University of Helsinki, 00014 Helsinki, Finland; Azrieli Faculty of Medicine, Bar-Ilan University, Safed 1311502, Israel; Department of Genetics, University of Groningen, University Medical Center Groningen, 9700 RB Groningen, the Netherlands; Department of Clinical Science, University of Bergen, 5021 Bergen, Norway; Department of Tropical Disease Biology, Liverpool School of Tropical Medicine, Liverpool, L3 5QA United Kingdom; Research Group for Child and Adolescent Health, Department of Clinical Medicine, Faculty of Health Sciences, UiT The Arctic University of Norway, 9037 Tromsø, Norway; Department of Paediatrics, Haydom Lutheran Hospital, 9001 Haydom, Mbulu -Manyara, Tanzania; Haydom Global Health Research Centre, Haydom Lutheran Hospital, 9001 Haydom, Mbulu -Manyara, Tanzania; Research Group for Child and Adolescent Health, Department of Clinical Medicine, Faculty of Health Sciences, UiT The Arctic University of Norway, 9037 Tromsø, Norway; Department of Paediatrics and Adolescence Medicine, University Hospital of North Norway, 9038 Tromsø, Norway; Department of Epidemiology, Johns Hopkins Bloomberg School of Public Health, Baltimore, MD 21205, United States; Microbiome Research Group, Medicine and Life Sciences Department, Universitat Pompeu Fabra, 08003 Barcelona, Spain; Centre for Ecological and Evolutionary Synthesis, Department of Biosciences, University of Oslo, 0371 Oslo, Norway; Department of Genetics, University of Groningen, University Medical Center Groningen, 9700 RB Groningen, the Netherlands; Department of Bacteriology and Immunology, Faculty of Medicine, University of Helsinki, 00014 Helsinki, Finland; Department of Preventive Medicine and Public Health, Food Science, Toxicology and Forensic Medicine, Faculty of Pharmacy and Food Sciences, University of Valencia, 46100 Burjassot, Valencia, Spain; Department of Biotechnology, Institute of Agrochemistry and Food Technology- Spanish National Research Council (IATA-CSIC), 46980 Paterna, Valencia, Spain; Global Health Institute, University of Antwerp, 2610 Wilrijk, Belgium; Department of Women’s and Children’s Health, Karolinska Institutet, 171 77 Solna, Sweden

**Keywords:** pregnancy, infant, microbiome, evidence gaps, research priorities and design, consensus

## Abstract

Early-life microbial exposures are essential for optimal development of human physiology. Yet, understanding of the human microbiome during pregnancy and childhood is still far from being complete. To identify knowledge gaps and establish research priorities, a multidisciplinary expert panel used the Delphi method for consensus development and conducted a literature search on early-life microbiome determinants. Responses from 55 researchers from an online survey were analyzed alongside keyword frequency from 20 501 publications. This approach enabled us to categorize existing evidence and highlight areas requiring investigation. While the main routes for mother-to-child bacterial transmission and their contributions to the newborn microbiome have been studied, many gaps remain. Priority areas include non-bacterial microbes, ecological principles of colonization, environmental and social influences, body sites beyond the gut, and factors affecting the maternal microbiome and its effects on the child’s microbiome. Significance of factors such as hygiene habits, non-antibiotic medications, and pollution remains to be uncovered. Knowledge is also limited on postnatal microbial sharing via household contacts and shared environments (e.g. family members, peers) and the contribution of these pathways to microbiome assembly. We hope this report will guide and inspire future research into the early-life microbiome as a modifiable factor in reducing disease risk.

## Teaser

The review highlights that, while early-life microbiome research has expanded rapidly, major evidence gaps remain, underscoring the need for broader, longitudinal, and more globally and clinically diverse studies.

## Introduction

The microbiome is a powerful regulator of human health and disease (Berg et al. 2020). Humans start acquiring their microbial companions principally at birth, but maternal microbes influence the next generation already during gestation (McDonald and McCoy 2019). During pregnancy, the gut microbiome carries out multiple functions, such as supporting pregnancy physiology, aiding fetal development, and ensuring optimal microbial transmission to the newborn (Koren et al. 2023). After birth, infants undergo intense physiological maturation, characterized by rapid development of metabolic, immune, and neuroendocrine systems. This maturation of the host physiology coincides with the gradual formation of the microbiome.

Ample research has demonstrated that disruptions to the microbiome, such as those caused by antibiotic exposure, alter signaling between the microbiome and the host, which is crucial for optimal physiological function. Perturbations of the early-life microbiome associated with increased risk of infectious (Miller et al. 2018, Shao et al. 2019, Shekhar and Petersen 2020) and non-communicable diseases (Dzidic et al. 2017, Stanislawski et al. 2018, Vatanen et al. 2018, Aversa et al. 2020), including early-life inflammatory diseases (Thänert et al. 2021, Chae et al. 2024). The wide-ranging effects of the microbiome on long-term health distinguish the early-life period, spanning from pregnancy to infancy, as a unique window for potential interventions to alter microbiome composition and reduce disease risks (Brodin, 2022)(Kaplan et al. 2011).

**Figure 1:**
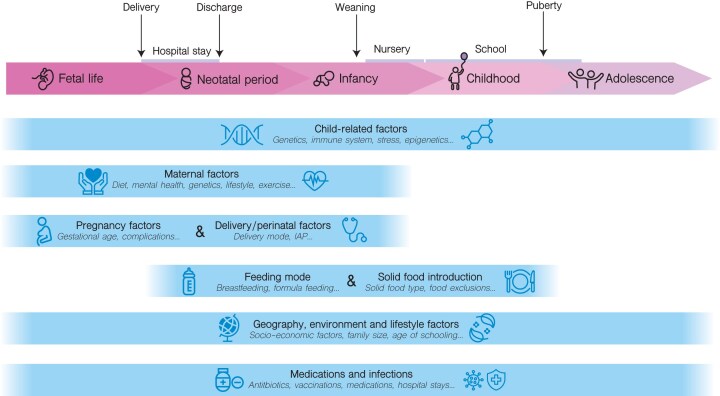
Determinants of human microbiome development. Illustration of known and suspected factors influencing human microbiome development from fetal life through adolescence. The timeline represents five developmental stages with key milestones: delivery, discharge (if born in hospital), weaning (i.e. introduction of complementary solid foods), nursery and school entry, and puberty. Horizontal bars indicate major determinant categories, including child biology (e.g. genetics, immune development, prematurity), maternal factors, pregnancy and delivery, nutrition, environmental and lifestyle exposures, and medications and infections. The length and position of each bar reflect the approximate timing and duration of influence for that factor group. IAP: intrapartum antibiotics.

Knowledge of the human microbiome during early life is thus central to strategies for disease prevention and treatment. By now, there is an understanding of some of the main factors that influence microbiome composition and function, including biological, environmental, and behavioral aspects (Fig. 1). Initially, the infant microbiome is influenced by the delivery mode, i.e. vaginal birth or cesarean delivery (Yassour et al. 2018), the mother’s microbiome and associated effects of diet (Rio-Aige et al. 2025) and medications such as peripartum antibiotics (Dubois et al. 2024, (Sinha et al. 2024), and early feeding practices, i.e. human milk, formula, or mixed feeding (Sela et al. 2008)(Ho et al. 2018). Following an individual’s growth, the microbiome continues to be shaped by a complex array of environmental exposures, diet, medication usage, lifestyle choices, as well as ecological and genetic factors (Falony et al. 2016, Zhernakova et al. 2016).

Despite recent progress, there are still significant gaps in our understanding of the human microbiome during pregnancy, infancy, and beyond (Robertson et al. 2019, Zhernakova et al. 2025). Evidence is lacking for many factors hypothesized to influence the composition and function of the human microbiome, such as non-antibiotic medications and pollution. Additionally, the interplay between different factors and their combined effects on the microbiome remains poorly understood. Moreover, as the field has rapidly expanded over the past two decades, studies have employed various designs, conduct, and even terminologies to describe aspects of the human microbiome, leading to heterogeneity and restricted applicability for translational research (Mirzayi et al. 2021, McGuinness et al. 2024, Porcari et al. 2025).

In earlier work by a subset of the present authors, ambiguous terminology in descriptions of human microbiome acquisition and transmission was addressed, alongside conceptual recommendations to support comparability (Rakoff-Nahoum et al. 2025). Here, we focus on mapping evidence gaps and defining priorities in early-life microbiome research. Our objective is to derive expert consensus and synthesize existing evidence on the determinants of the early-life microbiome, while identifying evidence gaps that will help establish research priorities. We present the results of our survey and literature searches, followed by their synthesis to pinpoint factors and sources that influence microbiome composition and function during early life. We then discuss which topics should be prioritized to benefit both research efforts and public health outcomes. The resulting report outlines evidence for a wide range of determinants of the early-life microbiome and highlights areas where knowledge gaps still exist.

## Methodology

Through a networking initiative facilitated by the Centre for Advanced Study at the Norwegian Academy of Science and Letters, 31 microbiome researchers joined the Young CAS Fellow 2022/2023 project “*Infant Gut Microbiome Acquisition: Off to a Healthy Start*”, focused on the early-life human microbiome. Participants included researchers from Europe, East Africa, West Asia, and North America, spanning PhD students to professors, with expertise in microbiology, bioinformatics, microbial ecology, epidemiology, pediatrics, medicine, genetics, and infection control. Fourteen of these researchers gathered at UiT The Arctic University of Norway (Tromsø, Norway) in August–September 2023 for a research sabbatical during which the basis of this report was developed.

### Consensus development

**Figure 2:**
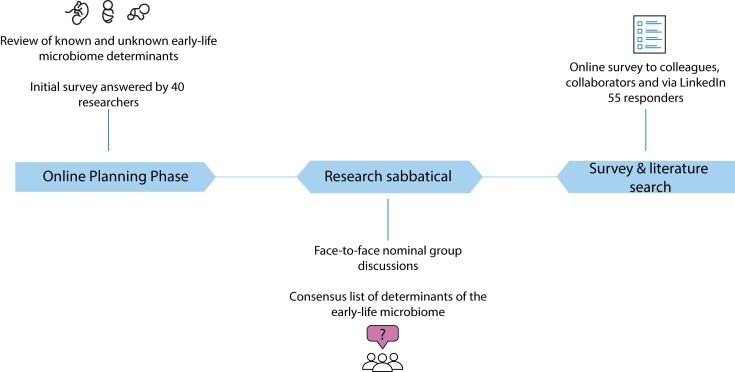
Project timeline.

To pinpoint knowledge gaps and research priorities in early-life microbiome research, we used the Delphi method (Dalkey and Helmer 1963, Khodyakov et al. 2023) and semi-structured group discussions. Our strategy included a preparatory survey followed by nominal group discussions (Murphy et al. 1998), with the aim of summarizing determinants of the early-life microbiome (Fig. 2). This followed a structured, iterative process for gathering and refining expert opinions towards a consensus, including a final survey and literature searches. A similar methodological approach has been used to establish a consensus on developing qualified microbiome-based biomarkers (Rodriguez et al. 2025) and microbiome testing in clinical practice (Porcari et al. 2025)

### Initial preparatory survey

During the planning phase with several online discussions (Fig. 2), participants of the sabbatical emailed the project coordinator research questions regarding the early-life microbiome that they considered the most pressing. This list was used to compile and test-run an initial preparatory survey with the overarching question, “*Which factors relevant for early life microbial transmission should be better studied*?”, and 20 sub-questions across three broad thematic areas (factors, sources, and mechanisms, see the original questionnaire in Supplementary PDF File 1). The survey included a five-point Likert scale, with the endpoints defined (1 = “well-studied” and 5 = “understudied”), while response options 2–4 were presented without labels. The additional options of “I don’t know” and free text were included for suggestions. This preparatory survey was emailed to ∼60 academics proposed by the participants, ranging from PhD students to principal investigators, of whom 40 anonymously responded (Supplementary Figure 1).

### Expert discussions

The frequency distribution of the responses served as a basis for structured face-to-face nominal group discussions conducted during the research sabbatical. One or two researchers first presented selected concepts, including potential controversies, in a 20-min presentation, followed by minuted discussions. We then compiled a list of determinants of the early-life microbiome, starting with a draft of terms provided by the presenting author(s). Selected terms were discussed and fine-tuned until a consensus was reached by all other authors, acknowledging associated limitations and challenges.

### Final expert survey

A final expert survey was conducted in June 2025, featuring nine thematic areas for grouping the determinants of early-life microbiome: 1) perinatal and obstetric factors; 2) infant-specific biological and developmental factors; 3) nutritional and dietary factors; 4) medical interventions and exposures; 5) environmental and lifestyle factors; 6) social and household exposures, 7) ecological and microbial dynamics/principles; 8) maternal factors; 9) different body sites. In addition to sending the survey link to our collaborators and colleagues, we also shared it on the LinkedIn social platform in various microbiome-related groups. This final survey collected responses from 55 respondents (see the original survey responses in Supplementary PDF File 2). Note that Supplementary Files 1 and 2 reproduce the preparatory survey and the final survey results as originally administered and generated; as such, they may contain typographical or informal wording that does not affect the interpretation of the data.

### Literature searches

Survey results were used to refine and prioritize the keyword lists for a structured literature mapping (last update: November 29, 2025). Using the survey-informed keywords, the literature mapping in Ovid MEDLINE® was based on keyword-frequency searches to estimate how commonly each thematic area is explored or mentioned in the early-life microbiome literature. This approach was intended to provide a broad bibliographic overview rather than being a traditional systematic review and therefore did not apply standard inclusion/exclusion criteria (e.g. language or publication year restrictions). All searches were based on the following combination of keywords: (“microbiome” or “microbiota”) AND (infant* or early life or neonat* or newborn* or toddler* or child* or teen* or adolescent*). The asterisk (*) is a truncation symbol and captures word variants, e.g. neonate(s), neonatal, neonatology. In the literature-mapping, we included the broader pediatric period (0–18 years) to quantify how research attention is distributed across age groups.

**Figure 3:**
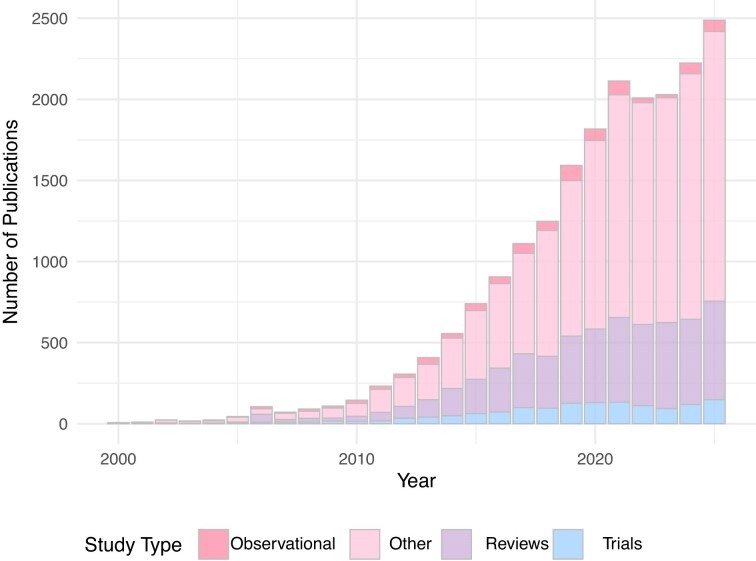
Annual number of publications indexed in Ovid MEDLINE®. Publications were retrieved using the following search strategy: “[(microbiome OR microbiota).ti,ab,kf. OR exp Microbiota/] AND [(infant* OR early life OR neonat* OR newborn* OR toddler* OR child* OR teen* OR adolescent*).ti,ab,kf. OR exp Infant/OR exp Pediatrics/OR exp Child/OR exp Adolescent/]”. Abbreviations and syntax: ti = title; ab = abstract; kf = keyword heading word; exp = “explode” [includes the selected Medical Subject Heading (MeSH) term and all narrower related terms]. The truncation symbol * retrieves word variants (e.g. neonat* captures neonate(s), neonatal, neonatology).

Medline sub-searches within those publications were then conducted to differentiate articles based on key words related to 1) sampling location; 2) age groups; 3) pediatric outcomes; 4) pregnancy and delivery; 5) maternal factors; 6) child-related factors; 7) nutrition, 8) environment; 9) medical exposures; 10) ecological mechanisms (Supplementary Excel File 3). For sampling-location sub-searches (e.g. urine and human milk), location terms were queried in combination with microbiome-related keywords (e.g. microbio*). In contrast, nutrition-related milk constituents that might be discussed without “microbiome” terminology (e.g. human milk oligosaccharides) were captured using separate, component-specific sub-searches. The search included title, abstract, keywords, and MeSH terms, yielding 20 501 publications. All retrieved records were imported into EndNote (v21), duplicates removed, and records with missing/incorrect bibliographic information (e.g. no author/title or erroneous publication year) excluded. Smart groups (EndNote’s dynamic collections that automatically group records based on predefined search rules) were created to mirror the Medline search strategy and sub-searches. For transparency and as a resource for readers, the resulting EndNote library (including bibliographic metadata and abstracts, but not full texts due to copyright and file-size constraints) is publicly available via OSF (www.osf.io/gp4dm). Percentages reported for thematic areas reflect all publication types retrieved by the search (including original studies, protocols, and reviews), except where explicitly stated otherwise (e.g. Fig. 3).

## Results and discussion

We mapped the current state of the early-life microbiome field by first defining terminology (informed by an initial preparatory survey), then conducting a final expert survey and a broad literature mapping to quantify term frequency. Together, these approaches highlighted knowledge gaps across perinatal/obstetric factors, diet and medical exposures, environmental and lifestyle influences, and ecological principles.

The survey featured a comprehensive overview of factors influencing the gut microbiome from pregnancy to infancy, highlighting the complexity of the early-life microbiome. We distributed the survey link to our colleagues and collaborators and also reached out to the broader scientific community via LinkedIn. Because we were targeting a specific audience, the survey had a strong academic and research-oriented demographic, with a majority actively engaged in or connected to research on the human microbiome (Supplementary Table 1). It was completed by 55 respondents, predominantly graduate students (*n* = 21), postdoctoral fellows (*n* = 11), and more senior researchers (*n* = 25), most of whom held professor, associate professor, or assistant professor positions. Respondents represented at least 23 institutions from 12 countries, including the United Kingdom, Finland, Belgium, Israel, Canada, the Netherlands, Spain, the United States, Norway, Sweden, Ireland, and Denmark. Twenty-two respondents did not specify either institution or country. Most respondents (*n* = 41, 75%) indicated that the early-life microbiome is their primary research interest. The answers thus represent a compiled perspective from a broad range of researchers (Supplementary PDF file S2), helping us to identify current gaps in understanding the early-life microbiome from pregnancy through childhood.

We next linked the expert opinions from the survey with current evidence by conducting a broad literature search (Supplementary Excel File 3). A total of 20 501 publications mentioned the keywords “microbiome” and “child” or their synonyms, representing a substantial body of literature on the early-life microbiome. The volume of research has been increasing over time, particularly since 2010, with a notable surge in annual publication rates in the last five years (Fig. 3). However, many studies were not categorized by study design in the Ovid Medline® database, limiting insights into the balance of evidence. Among those categorized by study design, review articles (N = 5250) outnumbered observational studies (N = 836) and clinical trials (N = 1403). This suggests a continued reliance on secondary reporting and analyses and idea generation/expert opinions rather than primary data generation, which is regarded as creating a higher level of scientific evidence. Similar trends have been reported in other areas of microbiome research (Hooks et al. 2019).

When stratified by age group, about one-third of the studies (30%) focused on infants (defined as 0 to 23 months), with newborns (0 to 1 month) alone accounting for 18% of the literature. By comparison, research on preschool-aged children (2–5 years) comprised 12% of studies, and children aged 6–12 years represented 24%. Adolescents (13–18 years) were mentioned in about 18% of publications. These findings indicate a concentration of research in the earliest phases of life, with decreasing attention given to older pediatric age groups. However, the apparent age gradient may partly reflect feasibility: stool sampling is often simpler in newborns and infants (e.g. via diapers) than in older children, where collection can be more logistically and ethically challenging.

In the following sections, we outline current evidence for five main categories of determinants, sources, and factors influencing the microbiome in early life: 1) maternal and infant factors; 2) nutritional factors; 3) medical factors; 4) environmental and lifestyle exposures; and 5) ecological principles. In this report, we use the term “early life” to encompass the period from pregnancy through infancy and childhood, with age ranges (e.g. newborns 0–1 month, infants 0–23 months, and older pediatric age groups) specified where relevant. A concluding discussion summarizes our interpretations for prioritizing future research areas.

## Maternal and infant factors

### Perinatal and obstetric factors

Perinatal factors are among the most studied determinants of early-life microbiome development, reflecting their central role in microbial transmission and early physiological programming. Human cohorts and experimental studies have demonstrated that gestational age and mode of delivery are strongly associated with infant microbiome composition, trajectories of microbial community assembly, and immune development (Fujimura et al. 2016, Chu et al. 2017, Korpela et al. 2018, Stokholm et al. 2018). In particular, preterm birth and cesarean delivery are consistently linked to delayed colonization by obligate anaerobes, altered or delayed bifidobacterial succession (Li et al. 2025), and increased abundance of facultative anaerobes and opportunistic taxa (Bokulich et al. 2016, Korpela et al. 2018, Shao et al. 2019).

**Figure 4:**
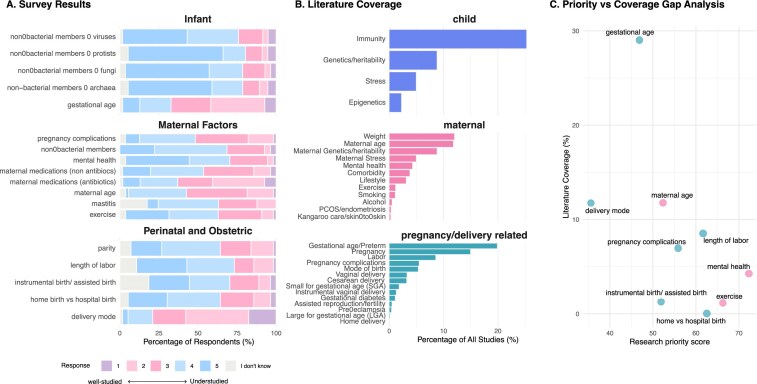
Maternal, infant, and perinatal determinants: expert survey ratings **(A)**, literature coverage **(B)**, and priority–coverage gaps **(C). (A)** Stacked bar charts showing the distribution of Likert scale responses (1 = well-studied, 5 = understudied) from survey respondents regarding which factors influencing the initial colonization of the infant’s microbiome are perceived as understudied and in need of further research. Responses are grouped by topic and normalized to sum to 100% per question. Grey segments represent “I don’t know” responses. **(B)** Horizontal bar charts showing literature coverage as the percentage of all retrieved publications that mention each keyword, grouped by topic (pregnancy/delivery related, maternal, child) and ordered by frequency within group. **(C)** Scatter plot comparing survey-derived priority scores (*x*-axis; weighted average of Likert responses excluding “I don’t know,” normalized to 0–100) with literature coverage (*y*-axis; % of publications mentioning the matched keyword). Points are colored by topic group. When a survey item mapped to multiple keywords, literature coverage was aggregated across the mapped keywords (note: keywords may overlap across publications).

In line with this knowledge, the literature mapping showed that gestational age and preterm birth are among frequently reported perinatal factors, with almost 20% of publications mentioning these determinants (Fig. 4). The keywords “pregnancy” and “labor” appeared in ∼15% and 9% of articles, respectively, while mode of birth was represented at a lower rate (5%), with “vaginal delivery” and “cesarean delivery/C-section” each mentioned in 3% of articles. In contrast, pregnancy complications were infrequently addressed: less than 6% of the literature mentioned such complications, with pre-eclampsia and gestational diabetes referred to in only 0.4% and 1.0% of papers, respectively. Other aspects, including home delivery, birth weight anomalies (small or large for gestational age), and assisted reproduction or fertility treatment, were addressed in fewer than 0.5% of publications.

When examined together with the expert survey, these findings reveal a contrast between perceived research needs and actual coverage in the published literature. This imbalance likely reflects a historical concentration of research effort on a limited set of perinatal exposures: gestational age, preterm birth, and mode of delivery have been central topics for decades, supported by large cohorts and clear associations with infant health outcomes (Dominguez-Bello et al. 2010, Fujimura et al. 2016, Stewart et al. 2018). By contrast, survey respondents emphasized that more granular perinatal factors remain understudied despite their likely relevance for microbiome acquisition and maturation, including length of labor (rated by 33% of responders as understudied), instrument assisted delivery (26%), place of birth (home vs hospital, 26%), and parity (20%). Qualitative responses further highlighted that available data on assisted births are currently too limited for robust statistical inference. Birth-related complications and maternal antibiotic intake before or during labor were also repeatedly identified as understudied, aligning with their low representation in the literature. Similarly, pregnancy complications, including pre-eclampsia and gestational diabetes, were rated by experts as relatively understudied compared with other perinatal factors. Overall, this suggests that systematic and sufficiently powered evidence across the full spectrum of perinatal exposures is still lacking.

### Maternal determinants

The maternal microbiome is increasingly recognized as a source of microbially derived metabolites and immune signals that mediate maternal–fetal interactions during pregnancy. Microbial metabolites and maternal antibodies induced by commensal and pathogenic microbes can cross the placenta and shape fetal development in utero (Thorburn et al. 2015, Gomez de Agüero et al. 2016, Apostol et al. 2020, Kimura et al. 2020, Brosseau et al. 2021). Animal studies further suggest that specific maternal microbial products, such as short-chain fatty acids, play a causal role in shaping offspring disease susceptibility (Thorburn et al. 2015, Kimura et al. 2020, Jama et al. 2024). However, in humans, the range, timing, and functional relevance of transplacental signals derived from the maternal microbiome remain largely unknown, pointing to a substantial room for future discovery. Consistent with this, our literature mapping suggests that many maternal determinants beyond broad characteristics remain comparatively underexplored. Overall, 18% of articles referred to general maternal characteristics, with maternal weight (12%), age (12%), and genetics or heritability (9%) being the most frequently mentioned traits. Maternal diseases, mental health, and stress were discussed in ∼4%, 4%, and 5% of publications, respectively, while lifestyle factors such as smoking, alcohol use, and physical activity appeared in fewer than 1% of studies. Notably, maternal endometriosis and kangaroo care (skin-to-skin contact) were rarely explored, accounting for <0.3% of publications.

Having summarized literature coverage of maternal determinants, we next return to the expert survey results to highlight which maternal factors were rated as comparatively understudied. Survey respondents identified several maternal factors as high-priority but insufficiently studied, including medications (antibiotic and non-antibiotic drugs, rated by 11% and 19% of responders as understudied, respectively), maternal mental health (41%), exercise (28%). Free text comments highlighted the need for more research on maternal diet, including supplements, pre- and probiotics, and timing of dietary changes before, during, and after pregnancy. Respondents also noted that although studies exist on mastitis and pregnancy complications, these are often limited by small sample sizes, constraining robust statistical analysis. A greater focus on postpartum maternal gut health was mentioned as particularly important, as this period is frequently omitted from birth cohorts despite its relevance for microbial transmission through close contact and breastfeeding. Strain tracking approaches were suggested as a tool to disentangle potential pathways of microbial sharing, including shared environment, direct contact, and breastfeeding (Valles-Colomer et al. 2023, Ferretti et al. 2025). In addition, paternal factors, including age, medications, lifestyle, and potential sperm-associated effects, were identified as significant research gaps, underlining that early-life microbiome research largely centers on maternal and infant factors and rarely captures the broader parental context, thereby overlooking potentially relevant contributors to early microbial assembly.

### Infant biological and developmental determinants

For child-related biological and developmental factors, terms pertaining to immune function were mentioned in 25% of the articles. On the other hand, genetics, stress, and epigenetic influences were mentioned in about 9%, 5%, and 2%, respectively. This illustrates a gap in research addressing the infant’s internal physiological or psychological state as a determinant of microbiome composition and *vice versa*. Host genetics has nonetheless emerged as a modest but biologically meaningful determinant of early-life microbiome variation. While adult heritability studies suggest that genetic effects are generally small relative to environmental influences (Rothschild et al. 2018, Gacesa et al. 2022), comparable large-scale efforts in infants are scarce. The most consistent evidence concerns the *FUT2* gene, whereby both maternal and infant secretor status influence infant gut microbiota composition, through loss-of-function variants that determine secretor versus non-secretor status and thereby regulate the availability of fucosylated glycans, including fucosylated human milk oligosaccharides, which selectively promote bifidobacterial colonization (Wacklin et al. 2011, Lewis et al. 2015, Korpela et al. 2018, Thorman et al. 2023). In mothers, this occurs via secretion of fucosylated human milk oligosaccharides that nourish specific taxa, whereas in infants, non-secretor status resulting from *FUT2* stop-gain mutations reduces mucosal fucosylation and is associated with lower abundance of bifidobacteria. Together, these findings indicate that although genetics exerts a narrower influence than perinatal and ecological factors, specific loci may nonetheless shape microbial trajectories throughout early infancy. Large-scale genome-wide association studies of neonatal microbiomes are anticipated to clarify the extent and mechanisms of these effects. Epigenetic influences, although linked to gut microbiome composition in adults (Demirkan et al. 2024), remain largely unexplored in infancy.

Finally, experts consistently emphasized the need to move beyond a bacteria-centric perspective. More than 50% of respondents rated viruses, protists, fungi, and archaea as understudied research topics, with bacteriophages highlighted as potentially central to understanding bacterial colonization dynamics. This stands in stark contrast to their limited representation in the current literature and underscores the need for methodological advances to better capture the full ecological complexity of maternal and infant microbiomes.

## Nutritional and medical factors

In addition to maternal and infant factors, nutrition and medical exposures are other key modulators of the early-life microbiome, acting through both direct microbial inputs and host-mediated physiological pathways (Bokulich et al. 2016, Amenyogbe et al. 2017, Zhang et al. 2024a). Diet provides the primary substrates shaping microbial succession after birth (Chu et al. 201 6), while medical interventions, including medications and preventive measures such as vaccination, can perturb microbial communities during sensitive developmental windows (Koenig et al. 2011, Hoskinson et al. 2024). Although these factors are widely recognized as critical for infant health and immune maturation, their effects on the microbiome have been studied unevenly, with a strong focus on a limited subset of exposures. This section continues with integrating expert survey responses with literature mapping to assess current evidence, identify imbalances in research coverage, and highlight priorities for future work in nutritional and medical determinants of the early-life microbiome.

### Nutritional and dietary determinants

**Figure 5:**
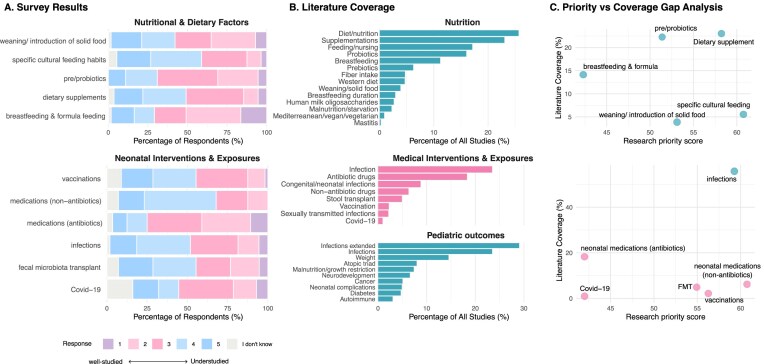
Nutritional and medical determinants: expert survey ratings **(A)**, literature coverage **(B)**, and priority–coverage gaps **(C). (A)** Stacked bar charts showing the distribution of Likert responses for how well each factor is currently studied in early-life microbiome research (1 = well-studied, 5 = understudied). Responses are normalized to 100% per item; “I don’t know” is shown in grey. Items are grouped into Nutritional & dietary factors and neonatal interventions and exposures. **(B)** Horizontal bar charts showing literature coverage for matched keywords, expressed as the percentage of all retrieved publications mentioning each keyword. Keywords are grouped into nutrition, medical interventions and exposures, and pediatric outcomes, and ordered by frequency within each group. **(C)** Scatter plots comparing survey-derived research priority scores (*x*-axis; weighted average of Likert responses excluding “I don’t know,” normalized to a 0–100 scale) with literature coverage (*y*-axis; % of publications mentioning the matched keyword). Each point corresponds to a survey item with one or more matched literature keywords; where multiple keywords were mapped to a single survey item, literature coverage was aggregated across the mapped keywords (keywords may overlap across publications).

The literature mapping of nutritional factors showed that general diet and nutrition and infant feeding or nursing practices were addressed in over 25% and 17% of articles, respectively (Fig. 5). Breastfeeding was mentioned in ∼11% of articles, however, breastfeeding duration appeared in only about 3% of publications. Probiotics and prebiotics were also relatively well represented (16% and 6%, respectively), whereas feeding transitions such as weaning or solid food introduction were mentioned in only 4% of studies. Dietary diversity and specific diet types (e.g. vegan, vegetarian, or diets from different global regions, and rural versus urban settings) were rarely explored, appearing in less than 1% of publications, while Western diets and fiber intake were each mentioned in ∼5% of studies. Malnutrition or starvation was addressed in about 2% of articles.

The expert survey partly reflected current literature but also revealed additional understudied topics related to nutrition. The respondents identified several priorities for further investigation, including specific cultural feeding habits (marked as understudied by 22% of responders), weaning or introduction of solid foods (20%), and dietary supplements (18%). Respondents further emphasized the need for a more nuanced understanding of feeding practices across cultures, particularly in non-Western settings. Qualitative feedback highlighted specific gaps, including the age at introduction of solid foods, diversity of foods introduced, patterns of complementary feeding (e.g. gradual introduction versus early high diversity), and the timing of allergen introduction. More frequent longitudinal sampling and the collection of highly detailed dietary data were repeatedly suggested as necessary to address these questions.

These findings highlight that many nutritionally relevant aspects remain insufficiently explored. Although breast- and formula-feeding (including duration) are commonly studied, knowledge remains limited regarding specific feeding practices and handling procedures, such as pumped versus directly fed milk and freshly expressed versus frozen human milk (mothers own or donor milk, from milk biobanks or stored home), and their effects on infant gut microbiome development. Survey respondents and prior studies also emphasized the importance of investigating specific human milk biomolecules, including oligosaccharides and glycosaminoglycans (chondroitin sulfate and dermatan sulfate), which are thought to play key roles in colonization by specific taxa (e.g. *Bacteroides* and *Bifidobacterium* spp.) and may represent targets for formula fortification or novel human milk fortification products.

Human milk itself represents a critical interface between maternal physiology and infant microbial colonization and therefore warrants particular attention (McCune et al. 2025). Beyond its nutritional components, the human milk microbiome has emerged as an important but poorly understood research frontier. Recent strain-resolved metagenomic work in mother–infant dyads supports that the human milk microbiome can contribute to infant gut community assembly and stability and may also share antimicrobial resistance genes within pairs, highlighting milk as both a nutritional and microbial exposure (Ferretti et al. 2025). Early work investigated breast-associated viruses in the context of cancer (Gross 1951), followed by amplicon sequencing studies comparing breast microbiota in cancer patients and healthy individuals (Urbaniak et al. 2014). Proposed origins of the milk microbiome include translocation from the maternal gut via immune cells, as well as contributions from maternal skin and the infant oral microbiome (Rodríguez 2014, Moossavi and Azad 2020), supported by evidence for an oral–entero–mammary transmission route (Zhong et al. 2022). However, only a limited number of studies have examined microbial communities in breast or mammary tissue outside lactation (e.g. in non-lactating individuals undergoing breast reduction or biopsy, (Urbaniak et al. 2014)), and evidence addressing the pre-pregnancy period remains scarce. These gaps likely reflect ethical, logistical, and technical barriers, including challenges in extracting sufficient microbial DNA from human milk samples with a low microbial biomass (Stinson et al. 2023). Fundamental questions regarding how the breast microbiome changes during pregnancy and lactation and how it contributes to infant gut seeding and immune maturation thus still remain incompletely addressed.

### Medical interventions and exposures

Medical exposures represent another major axis along which expert priorities and literature coverage diverge. In the literature mapping, pharmacological and infectious exposures were among the more frequently studied medical factors, but with a strong skew toward antibiotics. Antibiotic use was mentioned in ∼18% of studies, underscoring its well-established impact on the microbiome. Infectious diseases and congenital anomalies were also represented as medical conditions, together appearing in ∼9% of publications. In contrast, non-antibiotic medications were mentioned in only 6% of articles, stool transplants in 5%, vaccination in 2%, and more specific exposures such as COVID-19 (1%) or sexually transmitted diseases (2%) were rarely addressed. On the other hand, survey respondents identified vaccinations (marked as understudied by 20% of responders), infections (17%), and non-antimicrobial drugs (16%) as key areas requiring further investigation. Respondents also highlighted the role of hygiene practices and social isolation beyond the COVID-19 context, as well as the timing of infant vaccinations, noting that country-specific vaccination schedules may differentially influence gut microbiome development and immunomodulatory responses. Regarding microbiome-modulating interventions, fecal microbiota transplantation (FMT), which has been proposed as a strategy to restore microbiome composition in infants delivered by cesarean section (Korpela et al. 2020), was identified by 22% of survey respondents as an understudied area. However, one respondent commented on ethical and safety concerns regarding FMT in healthy infants, arguing that its use is controversial and should be limited to disease contexts where benefits outweigh risks, consistent with a “do no harm” principle.

Both literature search and survey answers, highlighted a striking lack of pediatric research on other medications known to affect the microbiome. Only a small number of studies have examined non-antibiotic drugs, primarily focusing on proton pump inhibitors negatively impacting the gastric acid barrier (Castellani et al. 2017, Brusselaers et al. 2022, Zhang et al. 2024b). This gap is notable given accumulating evidence from adult studies that many non-antibiotic drugs substantially alter microbiome composition (Le Bastard et al. 2018), and given that certain infant populations, such as preterm infants and those requiring neonatal intensive care, are frequently exposed to multiple non-antibiotic medications early in life. These drugs may influence the microbiome through diverse mechanisms (Grießhammer et al. 2025), including effects on gastrointestinal pH, motility, and cytotoxicity, yet this line of research has only minimally been extended to pediatric settings.

Taken together, the integrated analysis of survey data and literature mapping indicates that future research on nutritional and medical factors must move beyond broad binary categories (e.g. breastfeeding versus formula feeding, antibiotic use versus no antibiotic use). Instead, more nuanced, mechanistic, and longitudinal study designs are needed, incorporating molecular, microbial, clinical, and cultural dimensions. Promising directions include detailed investigations of human milk biomolecules and the milk microbiome (Ingram et al. 2024, Paone et al. 2024, Ferretti et al. 2025), systematic evaluation of vaccination timing and schedules (Wagner et al. 2025), and expanded study of non-antibiotic medications (Huang et al. 2024). Aligning future research with these priorities will be critical for advancing understanding of how diet and medical interventions shape early-life microbiome development and influence long-term health.

## Environmental and lifestyle exposures

**Figure 6:**
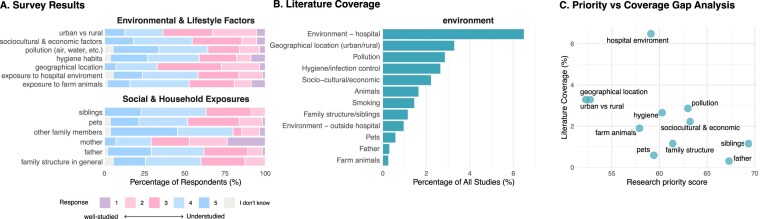
Environmental, lifestyle, and social exposures: expert survey ratings **(A)**, literature coverage **(B)**, and priority–coverage gaps **(C). (A)** Stacked bar charts showing the distribution of Likert scale responses assessing how well different environmental, lifestyle, and social factors influencing early-life microbiome are currently studied (1 = well-studied, 5 = understudied). Responses are normalized to 100% per item; “I don’t know” responses are shown in grey. Items are grouped into Environmental & lifestyle factors and Social & household exposures. **(B)** Horizontal bar charts showing literature coverage for matched keywords, expressed as the percentage of all retrieved publications mentioning each keyword. Keywords are grouped by environment-related themes and ordered by frequency within each group. **(C)** Scatter plot comparing survey-derived research priority scores (*x*-axis; weighted average of Likert responses excluding “I don’t know,” normalized to a 0–100 scale) with literature coverage (*y*-axis; percentage of publications mentioning the matched keyword). Each point represents a survey item with one or more matched literature keywords; where multiple keywords mapped to a single survey item, literature coverage was aggregated across mapped keywords (keywords may overlap across publications).

Environmental and lifestyle exposures represent a broad set of determinants that may shape early-life microbiome acquisition, yet both the expert survey and the literature mapping indicate that these factors remain comparatively underexplored (Fig. 6). Children encounter complex microbial inputs from their immediate surroundings, including household members, living environments (including nurseries, kindergartens, schools, etc.), hygiene practices, and wider socio-economic conditions, which together influence opportunities for microbial transmission and colonization. Unlike nutritional or medical factors, these exposures are often diffused, cumulative, and context-dependent, making them challenging to measure and compare across studies. As a result, environmental and lifestyle determinants have historically received less systematic attention in early-life microbiome research, despite growing recognition of their potential to shape microbial trajectories and health outcomes.

The literature mapping supports this perception, showing that environmental exposures are infrequently addressed relative to other domains. Hygiene practices and infection control were mentioned in ∼3% of articles, the hospital environment in 7%, and environmental location (e.g. rural versus urban settings) in about 3%. Pollution was mentioned in roughly 3% of publications, while family structure or sibling relationships appeared in only 1%. Paternal factors (0.4%), pets (0.6%), farm animal exposure (0.4%), and socio-cultural or economic context (2.3%) were among the least frequently referenced topics.

In the expert survey, pollution and hygiene habits were identified as top priorities for future research, with 29% and 24% of respondents rating them as understudied, respectively. Additional priorities included exposure to socio-cultural and economic factors (18%) and the hospital environment (18%). Responders also expressed a general consensus that microbial transmission from the environment, pets or animals, and non-maternal family members (e.g. grandparents and caregivers) is insufficiently studied. Several comments emphasized that while parity is often used as a proxy for sibling influence, studies rarely include sibling microbiomes as an explicit research focus, highlighting the need for designs that directly assess microbial contributions from different household contacts rather than relying on proxy measures. Together with the literature searches, these findings indicate that although environmental and lifestyle determinants are widely recognized as important, they remain peripheral in mainstream microbiome research.

### Social contact and microbial sharing

Beyond this quantitative underrepresentation, existing studies and conceptual work underscore the biological plausibility and potential importance of environmental microbial transmission. Recent strain-resolved analyses demonstrate that direct infant-to-infant microbial transmission contributes measurably to gut microbiome assembly, providing evidence that close contact and shared environments shape early microbial trajectories (Ricci et al. 2026). Family and cohabitation constitute another dimension of microbial exposure, as cohabiting family members and pets share a higher fraction of the gut microbiome than individuals in separate households, making them major contributors to infant colonization (Tavalire et al. 2021, Dubois et al. 2024). The presence of older siblings has been associated with increased microbiome diversity in infancy, likely reflecting both direct microbial sharing and indirect effects mediated through hygiene practices and culturally transmitted dietary and lifestyle habits (Laursen et al. 2015, 2017, Christensen et al. 2022). Beyond maternal seeding at birth, other caregivers and proximate individuals contribute to microbial acquisition, and even cross-generational transmission can be detected, albeit at low levels. In older individuals, gut microbiome strain sharing mirrors social networks, highlighting physical proximity as a key driver of microbial exchange (Brito et al. 2019, Tavalire et al. 2021, Amir et al. 2022, Valles-Colomer et al. 2022, 2023, Beghini et al. 2025). However, the health implications of these processes in early life remain poorly understood. In particular, it is unclear whether disease-associated (“dysbiotic”) microbiomes can be transmitted within households, and to what extent such transmission is modulated by shared diet and lifestyle.

### Household and built environment exposures

The geography and living environment of early life further shape microbial exposure. Infants born at home versus in hospitals, or those requiring prolonged neonatal intensive care, encounter markedly different microbial landscapes. Home births and family-centered care facilitate earlier exposure to microbes from close family members within and around the household environment, whereas hospital-based settings increase contact with disinfectants, infection control practices, antimicrobial-resistant species, and hospital-acquired pathogens (Combellick et al. 2018, Dong and Gupta 2019, Tauchi et al. 2019, Selma-Royo et al. 2024). Neonatal intensive care units themselves are shaped by feedback loops in which infant microbiomes seed the hospital environment and *vice versa* (Costeloe et al. 2016). Beyond clinical contexts, broader environmental features, including proximity to green spaces, farms, and animal contact (Thorsen et al. 2019, Depner et al. 2020, Buchholz et al. 2023), as well as social determinants such as environmental injustice and social deprivation (Batalha et al. 2025), and large-scale geographical differences between countries are likely to exert additional influences. Nevertheless, systematic studies linking these exposures to infant microbial trajectories remain sparse.

Taken together, our integrated analysis highlights a clear need to expand research beyond maternal and dietary determinants to more fully incorporate environmental, household, and social factors. Targeted, high-resolution studies are required to move beyond proxies such as parity and instead directly assess microbial contributions from siblings, caregivers, and pets, as well as from geographical and institutional contexts. Broader environmental exposures, including microplastics, endocrine disruptors, urbanization, and comparisons between rural and more natural environments, also warrant further investigation. Addressing these gaps will be essential for developing a holistic understanding of early-life microbiome development and its implications for lifelong health.

## Ecological and microbial dynamics

Assembly of the human microbiome is increasingly understood not only as a sequence of taxonomic changes, but as a dynamic ecological and evolutionary process in which microbial lineages are filtered by host context, competition, and adaptive capacity (Spor et al. 2011, Flores et al. 2025). While population-level succession patterns in infancy are conserved, individual trajectories vary markedly, reflecting the combined influence of stochastic processes (e.g. drift and priority effects) and deterministic forces such as host-mediated selection and microbe–microbe interactions. Recent conceptual frameworks emphasize that successful colonization depends on whether incoming microbial lineages can physiologically adapt to the rapidly changing infant gut environment (Sprockett et al. 2018, Debray et al. 2022, Ludington 2024), rather than solely on their arrival or abundance. This perspective shifts the focus from “who is present” to “why certain microbial cells persist,” highlighting microbial adaptation, functional plasticity, and ecological interactions as central principles shaping early-life microbiome development.

**Figure 7:**
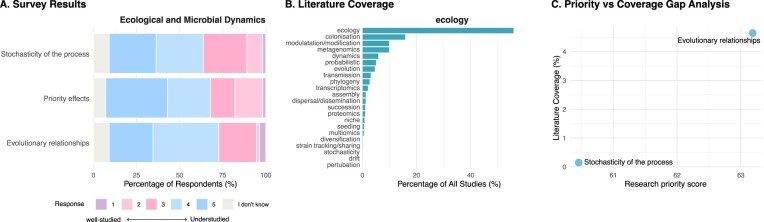
Ecological and microbial dynamics: expert survey ratings **(A)**, literature coverage **(B)**, and priority–coverage gaps **(C). (A)** Stacked bar charts showing the distribution of Likert scale responses assessing how well ecological and microbial dynamics processes relevant to early-life microbiome assembly are currently studied (1 = well-studied, 5 = understudied). Responses are normalized to 100% per item; “I don’t know” responses are shown in grey. Survey items include ecological concepts such as stochasticity, priority effects, and evolutionary relationships. **(B)** Horizontal bar charts showing literature coverage for matched ecology-related keywords, expressed as the percentage of all retrieved publications mentioning each keyword. Keywords encompass ecological processes (e.g. colonization, succession, transmission, drift, evolution), methodological approaches (e.g. metagenomics, multi-omics), and conceptual frameworks, and are ordered by frequency. **(C)** Scatter plot comparing survey-derived research priority scores (*x*-axis; weighted average of Likert responses excluding “I don’t know,” normalized to a 0–100 scale) with literature coverage (*y*-axis; percentage of publications mentioning the matched keyword). Each point represents a survey item matched to one or more literature keywords; where multiple keywords mapped to a single survey item, literature coverage was aggregated across mapped keywords (keywords may overlap across publications).

However, both the expert survey and the literature mapping indicated that ecological and evolutionary concepts remain insufficiently developed in early-life microbiome research (Fig. 7). Broad ecological terms such as “ecology” and “colonization” were frequently represented, appearing in 56% and 16% of publications, respectively, underscoring that foundational ecological concepts remain central in microbiome research. But more specific ecological and evolutionary terms were much less frequently mentioned, including “probabilistic” (5%), “transmission” (3%), “phylogeny” (3%), and “assembly” (1%). Emerging or methodologically demanding topics, such as dispersal or dissemination (1%), diversification (0.3%), strain tracking (0.2%), and stochasticity (0.1%), were addressed in only a small fraction of studies. This pattern suggests that while ecological framing is common, many mechanistic and process-oriented aspects of microbial ecology remain nascent or underrepresented in the literature.

The survey provides a complementary perspective. Priority effects, which describe how early colonizers modify resources and niches, thereby promoting or inhibiting the establishment of microbes that arrive later (Debray et al. 2022, Woelfel et al. 2024), were identified as understudied by 36% of respondents, while stochasticity of colonization, i.e. random variation in microbial establishment due to chance events during dispersal, survival, or initial growth, was highlighted by 27%. Evolutionary relationships between humans and their symbionts were also considered highly important (26%). Overall, respondents agreed that the ecological and evolutionary principles governing neonatal and infant gut colonization remain poorly understood. Qualitative comments emphasized that colonization success depends on a complex interplay between the environmental microbial pool, strain-level functional traits encoded in microbial genomes, and recipient-related factors such as host genetics, diet, exposome, and already established microbes. This complexity was highlighted as both a major challenge and a key opportunity for advancing the field.

When examined together, the survey and literature mapping reveal a mismatch between perceived importance and empirical coverage. Ecological and microbial dynamics were rated as the most understudied topic overall, with 46% of respondents identifying this domain as a priority. This strong consensus, coupled with the low representation of specific ecological processes in the literature, indicates that although the microbiome research community increasingly recognizes ecological and evolutionary principles as fundamental to early-life colonization, systematic and mechanistic investigations in this area remain limited.

The ecological perspective connects directly to evidence for a critical “window of opportunity” in early life, during which microbial colonization has long-term consequences for host physiology, especially immune development (Jain 2020, Busi et al. 2021). Experiments in germ-free animal models provide compelling support for this concept: the absence of microbial exposure during early life profoundly impairs immune system development, but these deficits can be fully corrected if commensals are introduced during the early postnatal period (Mazmanian et al. 2005, Al Nabhani et al. 2019). In infants, disruptions to microbiome establishment, such as those resulting from cesarean delivery, antibiotic use, or formula feeding, have been consistently linked to altered immune development and an increased risk of later developing allergic diseases (Pettersen and Arrieta 2020). At-risk infants often exhibit distinct microbial signatures, including depletion of specific beneficial taxa, fungal overgrowth, and altered metabolic activity, reinforcing the health relevance of early ecological processes. Cohort evidence also indicates that not only which microbes are acquired, but also the timing of acquisition, is critical for later health outcomes. For example, early-life colonization patterns across gut and respiratory niches, together with breastfeeding-related factors, predicted preschool asthma risk, further supporting the concept of time-sensitive windows in microbiome–immune development (Shenhav et al. 2024).

Despite strong evidence for a sensitive developmental window, the precise timing, sequence, and microbial signals required for optimal colonization remain poorly defined. Addressing these questions will require methodological innovation. High-throughput and high-resolution tools, including liquid chromatography–mass spectrometry for profiling proteins and metabolites combined with longitudinal DNA sequencing, have begun to reveal nuanced functional signatures of early-life microbiomes (Pettersen et al. 2022, Vatanen et al. 2022, Wu et al. 2024, Bargheet et al. 2025). However, substantial challenges remain in data integration, bioinformatics, and interpretation. Overcoming these hurdles will be essential for mapping the metabolic and ecological networks through which microbes influence infant physiology. In summary, ecological and evolutionary principles, such as priority effects, stochasticity, dispersal, and host–microbe coadaptation, are emerging as essential frameworks for understanding early-life microbiome assembly. Yet these principles remain understudied relative to their conceptual and clinical significance. Progress in this area will depend on integrating ecological theory with advanced experimental and analytical approaches to define how timing, microbial succession, and host interactions jointly shape lifelong health trajectories.

## Consolidated research priorities

Building on the domain-specific analyses above, we here integrate survey priorities and literature patterns to identify cross-cutting research gaps and strategic directions for the field.

### Body sites

The literature search revealed a strong bias toward studies involving fecal or gut microbiota, with more than half of all literature included (52%) mentioning fecal samples/stool. In contrast, relatively few articles examined other body sites. Vaginal and nasal microbiomes were mentioned in just over 3% of publications each, while oral or salivary microbiota were mentioned in 4% of the literature. Studies referring to the urogenital tract, often assessed using urine samples, accounted for ∼3% of publications, while human milk microbiome studies accounted for 2%. Papers on skin microbiome accounted for about 2%, and the endometrium was mentioned exceptionally rarely, at just 0.1%.

In the expert survey, the responders identified as high priority sites the gut microbiomes of infants and mothers (marked by 53% and 44%, respectively), followed by other family members (53%). Maternal vaginal microbiome (35%) and human milk (33%), as well as the oral cavity and skin of both mothers and infants (31% both), were also considered important for future studies. The metabolic profile of formulas was mentioned as important to include in future studies.

### Cross-domain themes

As described above, literature searches suggested that nutrition, birth mode, and antibiotics are the most frequently studied, while areas such as paternal influence, mental health, and socio-economic context remain underrepresented. When targeting pediatric health outcomes in the literature searches, the most common association explored in articles was between microbiome and infectious diseases, which was addressed in over 29% of the publications. Fewer publications explored links to the atopic triad (asthma, eczema, and allergic rhinitis; 8%), neurodevelopment (7%), diabetes (5%), or growth disorders (7%). Conditions such as cancer (5%) and autoimmune diseases (3%) were less frequently considered, suggesting that long-term disease outcomes remain an underexplored area in early-life microbiome research.

Moving to the expert survey responses on a general question of prioritizing research, 46% of respondents rated ecological and microbial dynamics/principles as the most understudied topic, followed by maternal factors (25%), infant factors (23%), and social and household exposures (23%). Several comments highlighted the relevance of many of the listed topics; however, it is also important to consider that different research questions will necessitate distinct study designs. For instance, the study of ecological and microbial dynamics will need denser sampling than some of the other topics. Finally, we received a suggestion for further investigation into the mechanisms behind maternal effects on the infant’s microbiome, including genetic similarities between mother and child, shared environments, breastfeeding, and whether functions enriched in the infant microbiome reflect the effects of maternal factors and corresponding microbiomes. The comment also proposed exploring the extent to which human milk components act as a bridge between the genetic influence behind maternal effects.

### Synthesis and forward-looking priorities

Across domains, several shared priorities emerge. First, there is a clear need to move beyond a bacteria-centric view of the microbiome. Both the survey and literature mappinng confirmed a major under-representation of non-bacterial members in microbiome research. While this bias has also been acknowledged by others (Heidrich et al. 2025) and is partly driven by technological limitations, it also reflects the well-established roles of bacteria in infant nutrition, immune maturation, and pathogen resistance. Bacteria are the most abundant and most metabolically active members of the infant gut microbiome, and current sequencing, culturing, and functional annotation tools are best optimized for bacterial communities. By contrast, non-bacterial components, including viruses (particularly bacteriophages), fungi, protists, and archaea, have received less attention, largely due to challenges in low-biomass detection, incomplete reference databases, and limited functional annotation. Expanding the focus to include these groups will be essential for a more holistic understanding of microbial community structure and function, but their relevance is unlikely to be uniform across taxa. For example, growing evidence suggests that bacteriophages play a central role in shaping bacterial colonization dynamics and ecosystem stability during infancy, whereas the ecological roles of archaea during the first year of life remain poorly resolved and may be highly context- and age-dependent. Progress will depend on targeted methodological advances, including improved viral and fungal enrichment strategies, long-read sequencing, strain-resolved metagenomics, and integrated multi-omics approaches, to determine which microbial groups exert meaningful effects on early-life development and health outcomes.

Second, diversification beyond the gut is needed to capture the spatial complexity of microbiome development better. The gut microbiome is currently the most studied, but other body sites of both the mother and the child, such as vaginal, nasal, oral, skin, and human milk microbiomes, remain relatively underexamined. Future research efforts should be diversified across different body sites to gain a more complete picture of the human microbiome’s development and its impact on localized and systemic health. However, we recognize that logistical and ethical challenges associated with invasive or uncomfortable sampling in children may contribute to this underrepresentation bias. Efforts, including home sampling and improved preservation methods, could help reduce the burden.

Third, respondents emphasized the importance of capturing microbiome dynamics across the full span of childhood. Their answers emphasized the need for longitudinal, high-frequency sampling frameworks to capture the dynamic nature of the microbiome beyond infancy. While some longitudinal follow-up studies are underway (Moraes et al. 2015, Lødrup Carlsen et al. 2018), the current research landscape predominantly focuses on the neonatal and infancy periods, leaving childhood, adolescence, and even young adulthood as “black boxes”. We acknowledge that large-scale microbiome collections have largely emerged as birth cohorts over the last 20 years, and it will take time to see the results of these longitudinal efforts and, consequently, the effect of early-life microbes on adult health. Still, there are already a few examples of follow-up in adolescents and young adults (Ahrens et al. 2024). We see a need for representative longitudinal studies in unselected (“healthy”) cohorts that span beyond the earliest phases of life, enabling comprehensive data collection to investigate correlations and interactions among various variables. Conversely, more research into “special” cohorts such as preterm neonates, children with peripartum complications, or neurodevelopmental issues is also warranted.

## Conclusions

This report maps determinants and conditions that may influence the early-life microbiome. We have identified current knowledge gaps by combining literature searches with expert opinions gathered through a Delphi methodology, which is well-suited to fields where evidence is fragmented and inconsistent. Although the early-life microbiome is increasingly recognized as a key regulator of future health and disease, the nuances of microbial acquisition and its long-term impacts remain incompletely understood. Current research is mainly focused on determinants with established effects on colonization, such as general nutrition, mode of birth, and antibiotic use. Future studies should broaden their scope to encompass underrepresented areas, including maternal health (e.g. mental and gynecological health, chronic diseases, exercise), non-antibiotic drug use, exposure to family members and the environment, socio-economic contexts, culture-specific nutritional habits, non-bacterial microbiota and their ecological dynamics, and microbiome-modulating interventions (Table 1). Addressing these priorities will require interdisciplinary approaches that integrate health, social, and environmental sciences to capture the complexity of microbiome development.

**Table 1:** Top 10 research priorities in early-life microbiome studies*.

Priority topic	Survey-derived priority [% respondents rating 4–5 on relevant item(s)]	Literature coverage (% publications)	Priority–coverage gap**	Why it matters
Ecological and microbial dynamics	46%	1–5%	High	Determines timing, persistence, and resilience of early microbial communities
Non-bacterial microbiome members	55%–62%	<2%	High	Key regulators of bacterial succession and ecosystem stability
Maternal mental health and psychosocial factors	41%	4–5%	High	May influence microbial transmission via stress, hormones, and caregiving
Maternal gut microbiome (including postpartum)	44%	<5%	High	Potential source of ongoing microbial transfer beyond birth
Infant–infant and household transmission	22%	<3%	High	Shapes microbial acquisition beyond maternal seeding
Specific birth-related factors (length of labor, instrument-assisted birth, home vs hospital)	30%	<1–5%	High	Poorly characterized determinants of early colonization patterns
Human milk composition beyond HMOs	33%	2%	Medium–high	Links maternal biology, nutrition, and infant microbial succession
Paternal factors	27%	<1%	High	Largely overlooked contributors to early microbial exposure
Body sites beyond the gut	33%	2%–4%	Medium–high	Relevant for localized immunity and systemic cross-site interactions
Long-term outcomes beyond infection	—	3%–7%	Medium	Needed to understand the lifelong consequences of early microbiome variation

*Table 1 synthesizes priorities across the final expert survey (Supplementary File 2; multiple items across thematic sections) and literature keyword coverage. “Survey priority” reflects the proportion of respondents rating the mapped item(s) as understudied (Likert 4–5). Where a priority topic spans multiple survey items, we report the maximum % rating 4–5 across mapped items; literature coverage was mapped to one or more keywords as described in the gap analysis methods.

**High/medium/low thresholds: priority = ≥40%/25%–39%/<25%; coverage = ≥10%/5%–9%/<5%. The gap category reflects discordance between priority and coverage (High gap = high priority + low coverage; medium gap = high priority + medium coverage or medium priority + low coverage).

To strengthen causal inference, the growing volume of research also necessitates improved reporting and categorization of study designs, supporting more prospective and experimental studies. Understanding initial microbiome acquisition remains central for identifying early windows of susceptibility and informing microbiome-targeted prevention strategies. At the same time, extending research into later childhood and adolescence is important to characterize longer-term microbiome trajectories, maturation, and resilience, and to identify additional windows during which exposures or interventions may influence health beyond the first 1000 days. Ultimately, coordinated analyses addressing specific research questions across multiple well-designed cohorts are needed to reveal robust, study-independent patterns that otherwise may be obscured within individual studies. Collaboration, open data practices that enable replication, and strict ethical and equity considerations will be essential to advance the field.

## Study strengths and limitations

By combining expert opinions with a review of over 20 000 publications, we provide a broad overview of the state of early-life microbiome research. The major strengths and uniqueness of our study lie in its breadth and systematic consensus process, which result in the identification of neglected areas. The survey drew responses from researchers at multiple career stages, thereby enhancing its representativeness. Yet, we do acknowledge that our results are likely biased towards the Global North. This relates in part to current global asymmetries in infrastructure and funding that shape where microbiome research is most commonly conducted. Although the broader project, during which this report was developed, included researchers from East Africa and other regions, the perspectives synthesized in this report are primarily derived from experts based in Europe and North America. Moreover, because a substantial share of survey respondents did not report their affiliation/country, the respondent geography cannot be fully characterized. Although several of us collaborate with researchers and clinicians in the Global South and have worked in these countries, we recognize that our experiences and research priorities may differ. We therefore emphasize the importance of global representation and collaboration in the (early life) microbiome field, particularly to study populations less exposed to Western lifestyles, and other populations in general, which have different priorities for health and research. Another limitation is that intermediate Likert-scale categories (2–4) were unlabeled in the survey. While this approach was chosen to avoid over-specification, it may have introduced variability in how respondents interpreted these intermediate scores and may limit comparability across respondents.

The study limitations are tied to database search restrictions, evidence biases, and the infancy of causal research in the field. While evidence synthesis methodology was applied through literature searches, it is important to acknowledge that our searches were intended to estimate the research attention or unexplored nature of certain topics. Because this mapping yielded a very large record set (>20 000), we did not perform screening, full-text review, or study-quality appraisal. We did not aim for a comprehensive and all-encompassing search as in traditional systematic or scoping reviews, which have specific research questions and in- and exclusion criteria to narrow down to a limited set of eligible studies. Our literature search relied on keyword, title, and abstract searches, which may misclassify whether factors were actively studied or merely mentioned. Moreover, we established that much of the existing evidence is skewed toward reviews rather than primary or interventional research. Therefore, more elaborate search strategies, including backward and forward citation tracking, are mandatory for a definitive understanding of specific topics, combined with pre-specified eligibility criteria. In addition, survey respondents were mostly academic specialists, under-representing perspectives from clinicians and policymakers. These constraints mean the study provides a valuable roadmap for future research, but should be interpreted with caution regarding the current depth and strength of the evidence base. We also acknowledge that studies addressing the identified priority areas may already be underway, and we will soon see evidence for some of the research priorities raised in this report.

## Supplementary Material

fuag010_Supplemental_Files
