# Supplementary material for: Off to a good start: current gaps and priorities in early-life microbiome research
Source: FEMS Microbiol Rev. 2026 Mar 9;50:fuag010. doi: 10.1093/femsre/fuag010 (PMC13044577; doi:10.1093/femsre/fuag010)
Supplement: fuag010_Supplemental_Files [file fuag010_supplemental_files.zip › Supplementary_File_Figures1_TableS1.pdf]

Supplementary Information for

Off to a good start: Current gaps and priorities in early-life microbiome research

By Pettersen et al.

Content:

Supplementary Figure 1. Results of the preparatory survey

Supplementary Table 1. Demographics of responders of the second, final survey

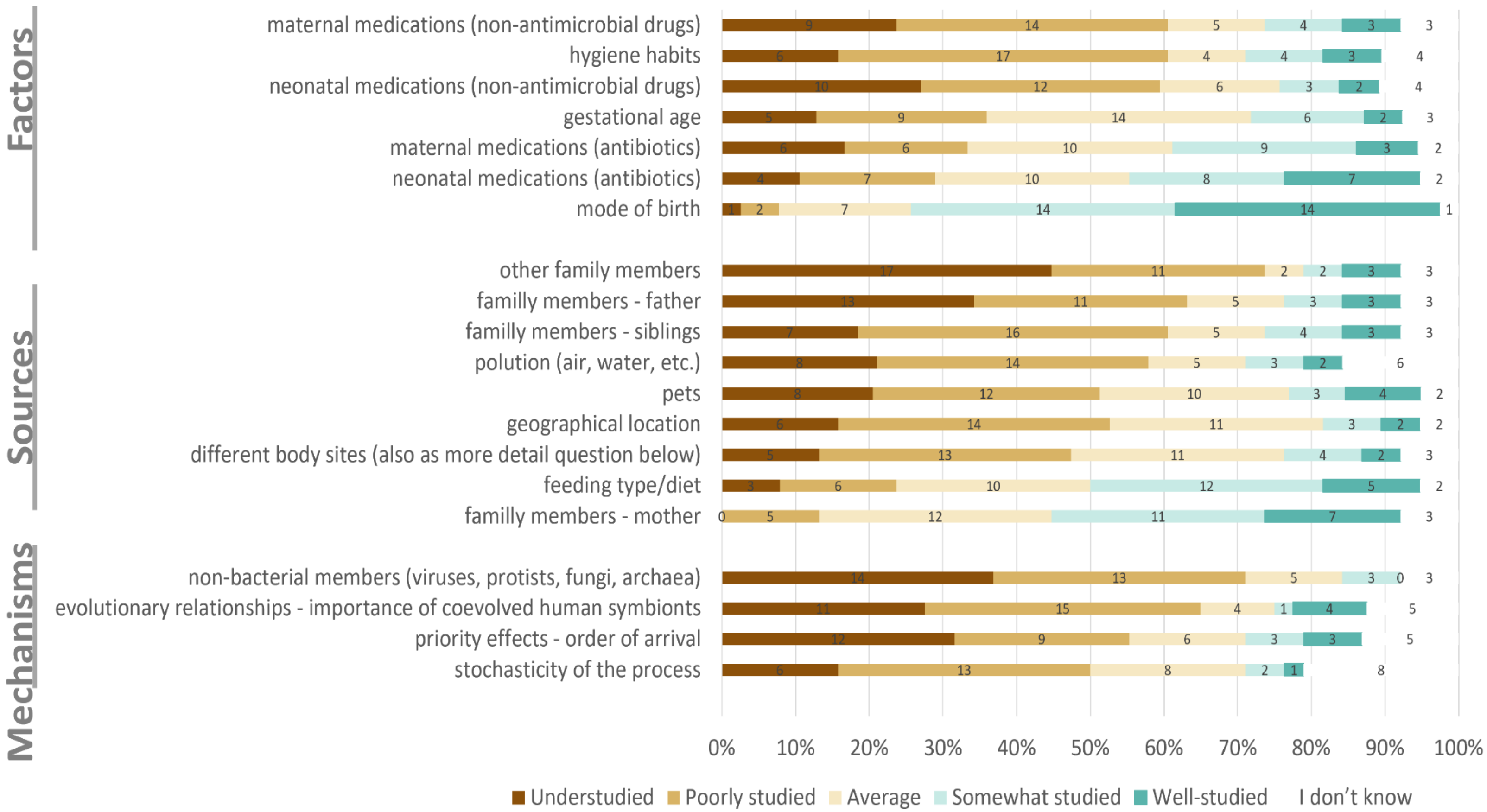

Supplementary Figure 1. A preparatory survey that received answers from 40 responders. Compiled bar charts illustrating the distribution of answers to the question, “Which factors relevant for early life microbial transmission should be better studied?”. Note that some sources might also act as factors, highlighting the dynamic and multifactorial nature of the gut microbiome

Supplementary Table 1. Demographics of responders of the second, final survey. Note that the initial, preparatory survey did not contained questions on reponders demograpphics.

Domain	Category	n	%
Career stage / role (select-one)	Graduate student (MSc/PhD)	21	38.2
	Postdoctoral fellow	11	20
	Assistant Professor	6	10.9
	Associate Professor	7	12.7
	Professor	7	12.7
	Research staff scientist (research institute)	4	7.3
	Research staff scientist (higher education)	1	1.8
	Undergraduate student	1	1.8
	Industry research staff scientist	0	0
	Other	2	3.6
Connection to early-life microbiome (select-all)	Primary research interest	41	74.5
	Overlaps with main research field	14	25.5
	Curiosity	4	7.3
	Have heard about it, but don't know much	1	1.8
	Other	0	0
Country of main employment (free-text; derived)	Finland	6	10.9
	Netherlands	6	10.9
	Sweden	5	9.1
	Spain	5	9.1
	Norway	3	5.5
	United Kingdom	2	3.6
	Belgium	2	3.6
	Israel	1	1.8
	United States	1	1.8
	Ireland	1	1.8
	Denmark	1	1.8
	Unclear / not stated (institution only or ambiguous)	22	40
