# Supplementary material for: Off to a good start: current gaps and priorities in early-life microbiome research
Source: FEMS Microbiol Rev. 2026 Mar 9;50:fuag010. doi: 10.1093/femsre/fuag010 (PMC13044577; doi:10.1093/femsre/fuag010)
Supplement: fuag010_Supplemental_Files [file fuag010_supplemental_files.zip › Supp_File1_PreparatorySurvey-June23.pdf]

Nettskjema

Consensus development on microbial transmission during early life - initial questionnaire

One aim of this work is to clarify the terminology when describing microbial transmission in early life. Many terms are found in literature, including vertical and horizontal transmission, strain sharing, and intra-generational transmission, but the exact meaning of these words remains vague.
The second objective is to summarize the main factors influencing microbial transmission.
With this initial questionnaire, we are reaching out to a broader community of microbiome researchers to identify terms and factors related to microbial transmission. We would appreciate if you can give us feedback on the following three questions. The survey is anonymous.

Terminology on microbiome transmission

Q1 - Which terms do you reckon need a definition?

The scale represents a range for a term being not defined at all (1) to very well-defined (5).

vertical transmission

1
2
3
4
5
I don’t know

horizontal transmission

1
2
3
4
5
I don’t know

co-acquisition

1
2
3
4
5
I don’t know

microbial/strain sharing

1
2
3
4

5

I don’t know

strain meaning in metagenomics

1

2

3

4

5

I don’t know

intragenerational microbial transmission

1

2

3

4

5

I don’t know

intergenerational microbial transmission

1

2

3

4

5

I don’t know

seeding

1

2

3

4

5

I don’t know

If you have suggestions for other relevant terms that might need a definition, please let us know!

Factors affecting microbial transmission

Q2 - Which factors relevant for early life microbial transmission should be better studied?

Note that the factors listed below are relevant both for "early life microbiome" in general and for "early life microbial transmission".We are specifically asking about factors relevant to microbial transmission. The scale represents a range of a factor being understudied (1) to well-studied (5).

Nettskjema

mode of birth

1
2
3
4
5
I don’t know

gestational age

1
2
3
4
5
I don’t know

feeding type/diet

1
2
3
4
5
I don’t know

maternal medications (antibiotics)

1
2
3
4
5
I don’t know

maternal medications (non-antimicrobial drugs)

1
2
3
4
5
I don’t know

neonatal medications (antibiotics)

1
2
3

4
5
I don’t know

neonatal medications (non-antimicrobial drugs)

1
2
3
4
5
I don’t know

familly members - mother

1
2
3
4
5
I don’t know

familly members - father

1
2
3
4
5
I don’t know

familly members - siblings

1
2
3
4
5
I don’t know

other family members

1
2
3
4
5
I don’t know

pets

1
2
3
4
5
I don’t know

different body sites (also as more detail question below)

1
2
3
4
5
I don’t know

non-bacterial members (viruses, protists, fungi, archaea)

1
2
3
4
5
I don’t know

evolutionary relationships - importance of coevolved human symbionts

1
2
3
4
5
I don’t know

geographical location

1
2
3
4
5
I don’t know

stochasticity of the process

1
2
3
4

5
I don’t know

priority effects - order of arrival

1
2
3
4
5
I don’t know

polution (air, water, etc.)

1
2
3
4
5
I don’t know

hygiene habits

1
2
3
4
5
I don’t know

If you have suggestions for other factors, please let us know!

Q3 - What body sites should be sampled in future cohorts?

The scale represents a range for a body site importance from no need to be sampled (1) to necessary to be sampled (5).

Gut - mother, C-section

1
2
3
4
5
I don’t know

Oral cavity - mother, C-section

1
2
3

4
5
I don’t know

Skin - mother, C-section

1
2
3
4
5
I don’t know

Vagina - mother, C-section

1
2
3
4
5
I don’t know

Gut - mother, vaginal delivery

1
2
3
4
5
I don’t know

Oral cavity - mother, vaginal delivery

1
2
3
4
5
I don’t know

Skin - mother, vaginal delivery

1
2
3
4
5
I don’t know

Vagina - mother, vaginal delivery

1
2
3
4
5
I don’t know

Gut - health personnel

1
2
3
4
5
I don’t know

Oral cavity - health personnel

1
2
3
4
5
I don’t know

Skin - health personnel

1
2
3
4
5
I don’t know

If you have suggestions for other body sites, let us know!
