# Supplementary material for: Off to a good start: current gaps and priorities in early-life microbiome research
Source: FEMS Microbiol Rev. 2026 Mar 9;50:fuag010. doi: 10.1093/femsre/fuag010 (PMC13044577; doi:10.1093/femsre/fuag010)
Supplement: fuag010_Supplemental_Files [file fuag010_supplemental_files.zip › Supp_File2_FinalSurvey-June25.pdf]

NETTSKJEMA

Report

Priorities for early life microbiome research - questionnaire

Downloaded: 15 January 2026 at 17:11

Contents

Which of the factors that influence the initial colonization of the infant's microbiome should be better studied? 3

If you have comments or suggestions, please let us know! 3

Which factors relevant to the early life microbiome should be better studied? 4

If you have comments or suggestions, please let us know! 4

Which factors relevant to the early life microbiome should be better studied? 5

If you have comments or suggestions, please let us know! 5

Which factors relevant to the early life microbiome should be better studied? 6

If you have comments or suggestions, please let us know! 6

Which factors relevant to the early life microbiome should be better studied? 7

If you have comments or suggestions, please let us know! 7

Which factors relevant to the early life microbiome should be better studied? 8

If you have comments or suggestions, please let us know! 8

Which factors relevant to the early life microbiome should be better studied? 9

If you have comments or suggestions, please let us know! 9

Which maternal factors relevant to the early life microbiome should be better studied? 10

If you have comments or suggestions, please let us know! 10

What body sites should be sampled in future cohorts? 11

If you have comments or suggestions, please let us know! 12

Which factors should be prioritised to study in future microbiome studies? 12

If you have comments or suggestions, please let us know! 13

What is your experience in the microbiome field? 14
If you choose "other" please specify. 14
Please let us know the institution and country of your main employment. 14 - 15
What is your connection to the early life microbiome (maternal microbiome during pregnancy and the infant microbiome)? 16
If you choose "other" please specify. 16

Thank you for considering participating in our survey!
Our goal is to create a comprehensive overview of factors that influence the gut microbiome from pregnancy to infancy. To gather a wide range of insights, we are distributing this anonymous questionnaire to the broader microbiome research community.
Your input will help us map the current knowledge on the sources and factors affecting the early life microbiome, with a specific focus on the maternal microbiome during pregnancy and the infant microbiome. We appreciate your feedback on the following topics:

- Perinatal and Obstetric Factors
- Infant-Specific Biological and Developmental Factors
- Nutritional and Dietary Factors
- Medical Interventions and Exposures
- Environmental and Lifestyle Factors
- Social and Household Exposures
- Ecological and Microbial Dynamics/Principles
- Maternal Factors
- Different body sites

I. Perinatal and Obstetric Factors (Mode of Birth & Immediate Post-Birth)

Which of the factors that influence the initial colonization of the infant's microbiome should be better studied?

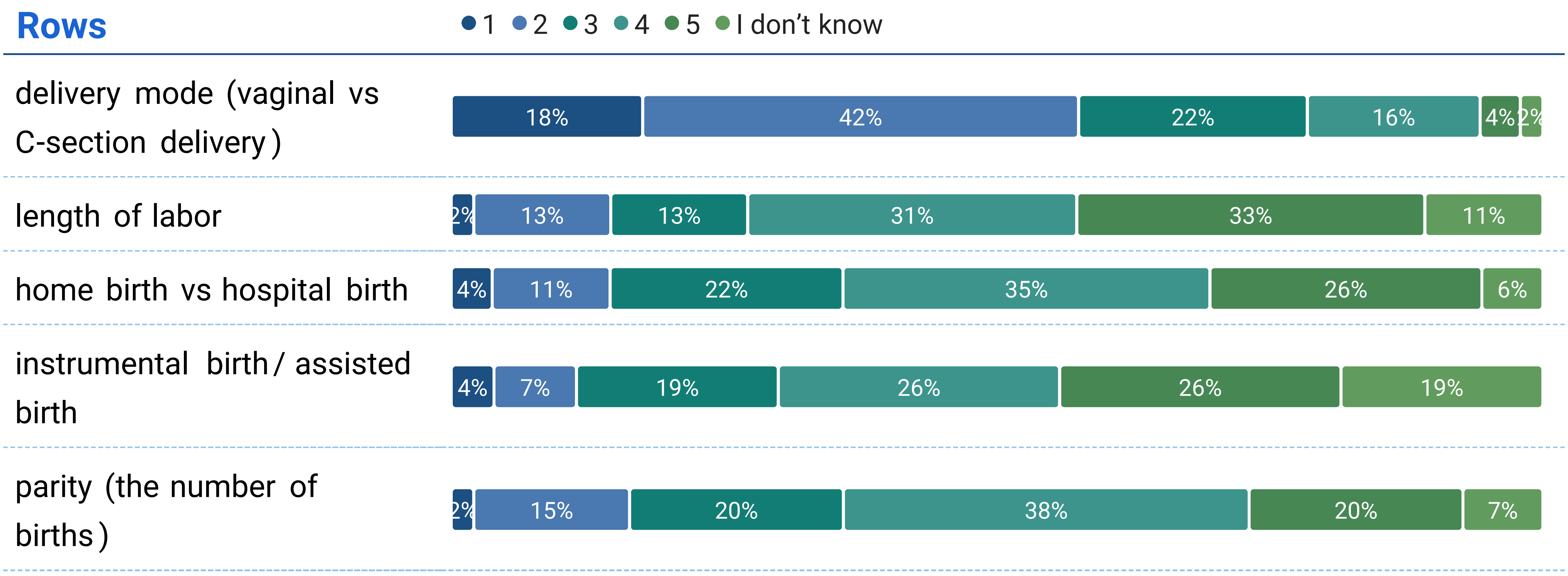

Rows	1	2	3	4	5	I don't know
delivery mode (vaginal vs C-section delivery)	10	23	12	9	2	1
length of labor	1	7	7	17	18	6
home birth vs hospital birth	2	6	12	19	14	3
instrumental birth/ assisted birth	2	4	10	14	14	10
parity (the number of births)	1	8	11	21	11	4

If you have comments or suggestions, please let us know!

Number of submissions: **4**

Submissions

Transmission of microbes from the environment, pets/animals and from other people, especially family members (other than maternal) and daycare peers is understudied. Strain level resolution is highly needed in these types of studies.

As of now the number of assisted birth data in literature is quite small to do any kind of statistics based inference. Although, c-sec birth seems to have low virome association atleast till 4 months of time, this needs lot of samples to be screened for meaningful

[illegible]
comparisions. This holds true for the other parameters listed above.

another aspect that is currently understudied is the impact of maternal antibiotic intake pre- and during labour on the infant microbiome assembly and development

Birth-related complications

II. Infant-Specific Biological and Developmental Factors

Which factors relevant to the early life microbiome should be better studied?

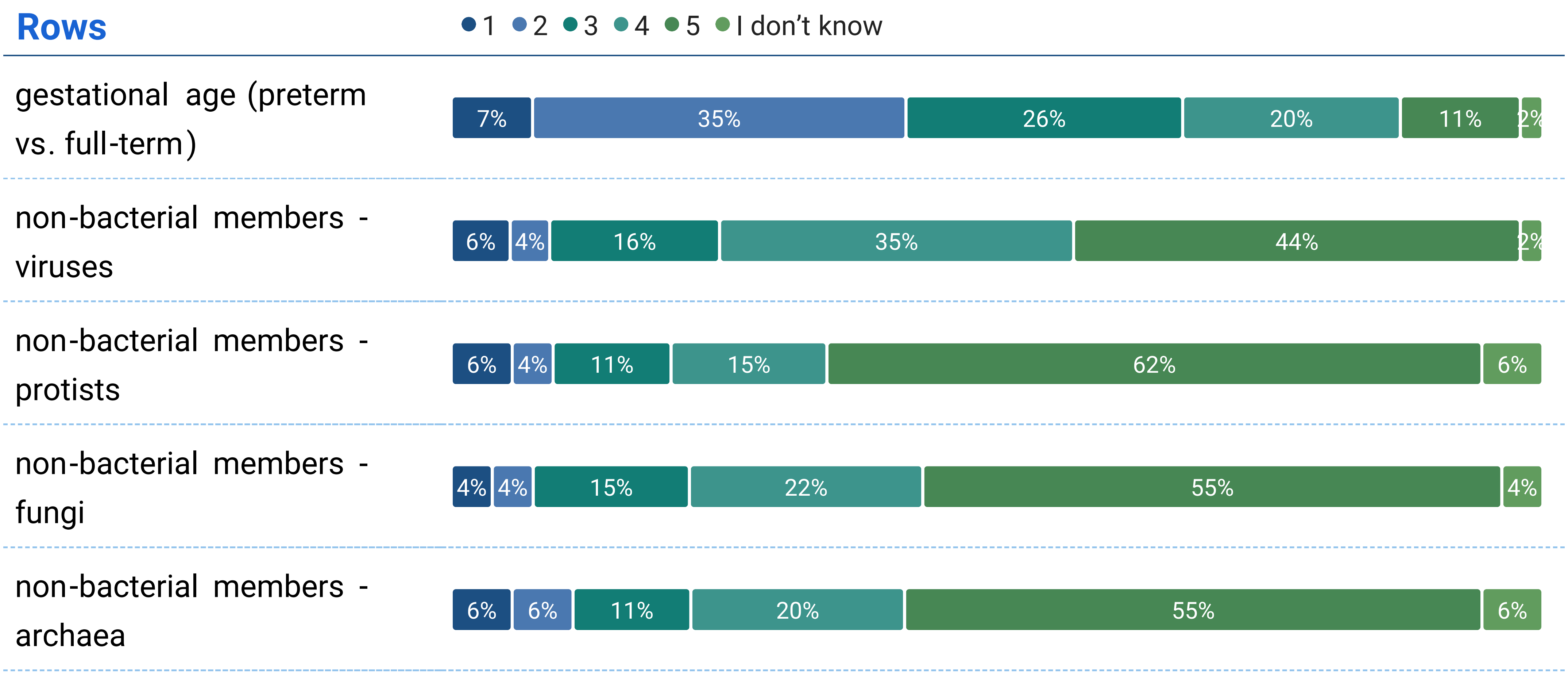

Rows	1	2	3	4	5	I don't know
gestational age (preterm vs. full-term)	4	19	14	11	6	1
non-bacterial members - viruses	3	2	9	19	24	1
non-bacterial members - protists	3	2	6	8	34	3
non-bacterial members - fungi	2	2	8	12	30	2
non-bacterial members - archaea	3	3	6	11	30	3

If you have comments or suggestions, please let us know!

Number of submissions: **1**

Submissions

All other members, than bacteria, of the early life microbiome are understudied. Bacteriophages are likely of key importance for understanding bacterial colonization dynamics.

III. Nutritional and Dietary Factors

Which factors relevant to the early life microbiome should be better studied?

Rows	1	2	3	4	5	I don't know
specific cultural feeding habits	4%	9%	29%	33%	22%	6%
weaning / introduction of solid food	7%	29%	24%	22%	20%	2%
breastfeeding & formula feeding (including duration)	16%	35%	20%	13%	15%	2%
pre/probiotics	6%	26%	38%	20%	11%	
dietary supplements	6%	9%	36%	27%	18%	4%

Rows	1	2	3	4	5	I don't know

specific cultural feeding habits	2	5	16	18	12	3
weaning / introduction of solid food	4	16	13	12	11	1
breastfeeding & formula feeding (including duration)	9	19	11	7	8	1
pre/probiotics	3	14	21	11	6	0
dietary supplements	3	5	20	15	10	2

If you have comments or suggestions, please let us know!

Number of submissions: **6**

Submissions

Impact of age of introduction of solids, diversity of foods introduced, patterns of complementary food introduction (slowly introducing food items or starting with a high diversity), timing of introduction of food allergens, specific cultural feeding habits are all greatly understudied questions.

The effects on the gut microbiome of introduction of specific foods during complementary feeding is still poorly understood. High longitudinal sampling frequency and high resolution in dietary information is necessary.

The non european diets are super interesting

While breastfeeding and formula feeding (including duration) has been well studied, it could be of interest to study the components of breast and formula milk and how they may contribute to gut microbiome development, especially formula milks fortified with HMOs and/or pro-bioitcs.

I would focus on breastmilk oligosaccharides, specifically the presence and concentration of chondroitin and dermatan sulfate, as they seem to be necessary for Bacteroides colonisation in early life.

Pumped breat milk vs direct breastfeeding vs formula

IV. Medical Interventions and Exposures

Which factors relevant to the early life microbiome should be better studied?

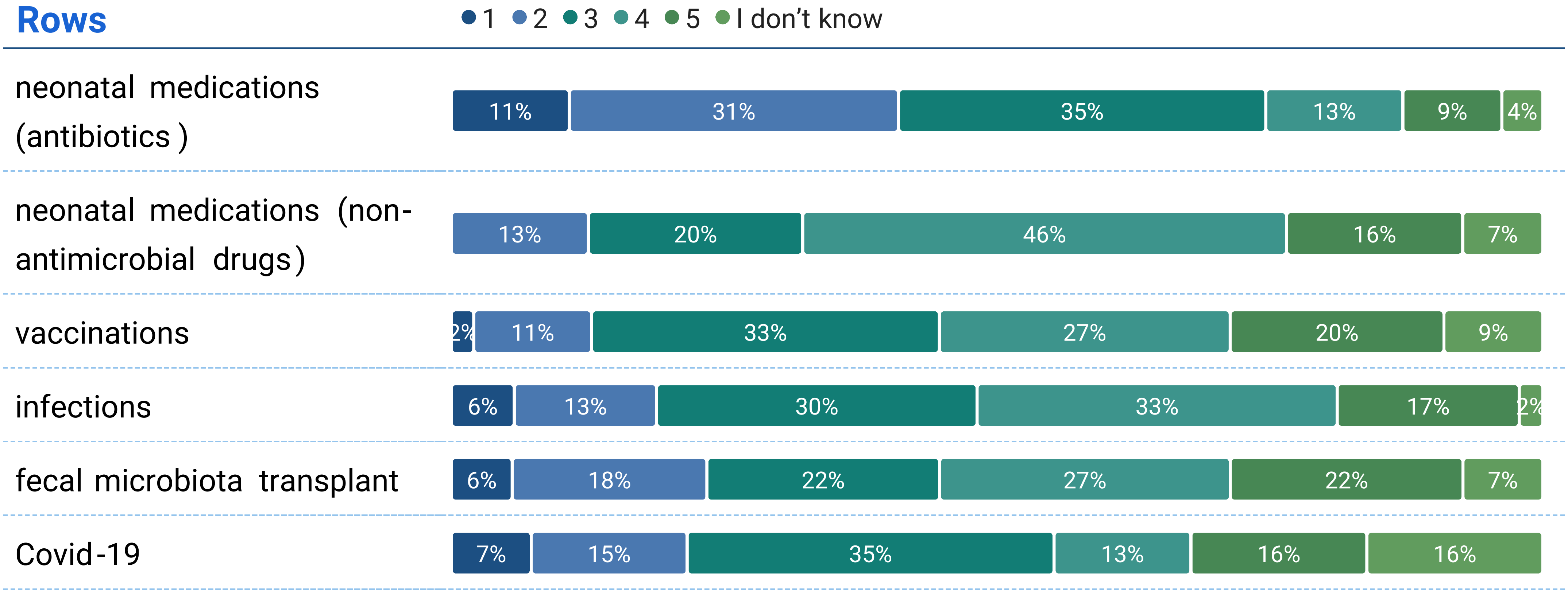

Rows	1	2	3	4	5	I don't know
neonatal medications (antibiotics)	6	17	19	7	5	2
neonatal medications (non-antimicrobial drugs)	0	7	11	25	9	4
vaccinations	1	6	18	15	11	5
infections	3	7	16	18	9	1
fecal microbiota transplant	3	10	12	15	12	4
Covid-19	4	8	19	7	9	9

If you have comments or suggestions, please let us know!

Number of submissions: **3**

Submissions

Not only COVID-19 infections but impact of social isolation and hygiene practices has not been well studied except for a couple of studies.

The moment when the baby starts being vaccinated is an interesting topic. While in some countries, babies can be vaccinated against many diseases very early after delivery, here in Spain, for example, it seems to be delayed by three months (I suppose). Is it possible that this window changes something in terms of immunomodulation by the gut microbiome and

then affects immunisation after vaccination?

I did not rank FMT as FMTs on healthy infants is an extremely controversial practice, bordeline against the "do no harm" principle. Due to the risks, it should be considered only in diseased subjects where other treatments fail. I do not believe research on FMT in health infants should be studied more or less, but rather halted altogether until deeper understanding of the process is provided by FMT studies in diseases subjects.

V. Environmental and Lifestyle Factors

Which factors relevant to the early life microbiome should be better studied?

Rows	1	2	3	4	5	I don't know
geographical location (country, continent)	4%	24%	40%	26%	7%	
urban vs rural	6%	27%	31%	24%	13%	
pollution (air, water, etc.)	4%	13%	20%	31%	29%	6%
hygiene habits (e.g., handwashing, cleaning practices, sanitization)	9%	9%	24%	33%	24%	4%
exposure to farm animals	9%	11%	29%	38%	15%	2%
socio-cultural and economic factors	6%	9%	31%	36%	18%	
exposure to hospital enviroment	2%	15%	26%	35%	18%	6%

Rows 1 2 3 4 5 I don't know

geographical location (country, continent)	2	13	22	14	4	0
urban vs rural	3	15	17	13	7	0
pollution (air, water, etc.)	2	7	11	17	16	3
hygiene habits (e.g., handwashing, cleaning practices, sanitization)	5	5	13	18	13	2
exposure to farm animals	5	6	16	21	8	1
socio-cultural and economic factors	3	5	17	20	10	0
exposure to hospital enviroment	1	8	14	19	10	3

If you have comments or suggestions, please let us know!

Number of submissions: **0**

This question has no answers

VI. Social and Household Exposures

Which factors relevant to the early life microbiome should be better studied?

Rows ● 1 ● 2 ● 3 ● 4 ● 5 ● I don’t know

family structure in general

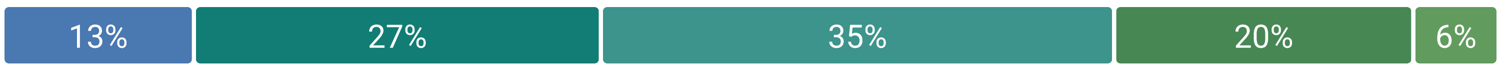

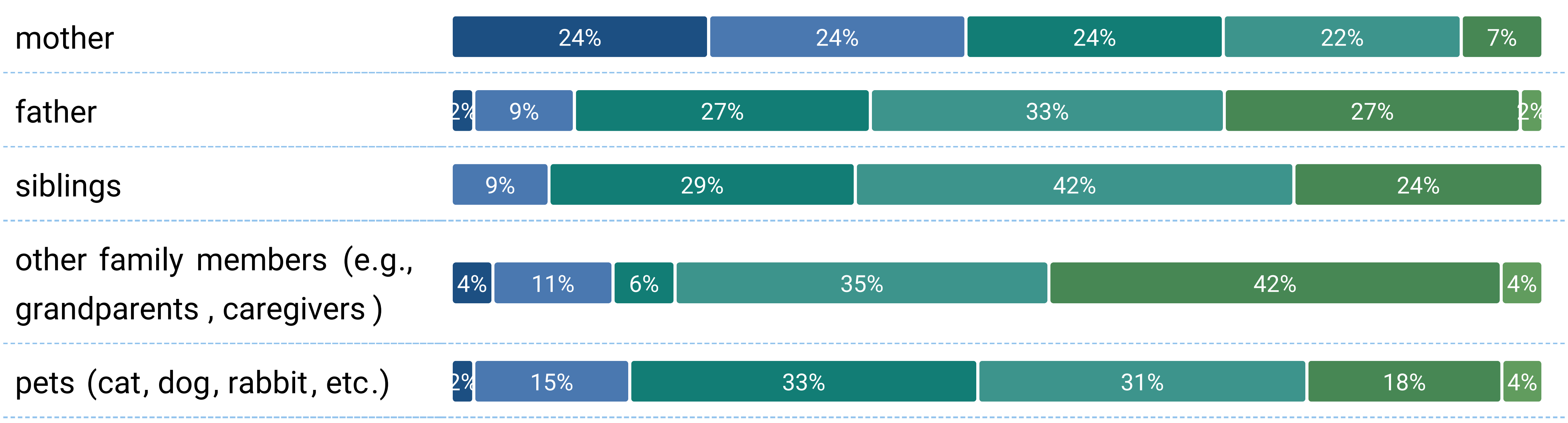

Rows	1	2	3	4	5	I don't know
family structure in general	0	7	15	19	11	3
mother	13	13	13	12	4	0
father	1	5	15	18	15	1
siblings	0	5	16	23	13	0
other family members (e.g., grandparents , caregivers)	2	6	3	19	23	2
pets (cat, dog, rabbit, etc.)	1	8	18	17	10	2

If you have comments or suggestions, please let us know!

Number of submissions: **1**

Submissions

While parity has been used as a proxy to investigate the relations of having a sibling on the infant gut microbiome, as far as I am aware, the inclusion of sibling gut microbiomes is usually not the main aim of studies. Rather they are included because of longitudinal study designs, which by chance, would include siblings or twins with the sample size being much smaller compared to singletons.

VII. Ecological and Microbial Dynamics/Principles

Which factors relevant to the early life microbiome should be better studied?

Rows ● 1 ● 2 ● 3 ● 4 ● 5 ● I don’t know

Stochasticity of the process (randomness in initial colonization and subsequent changes)

Priority effects - order of arrival (the impact of early colonizers on subsequent microbial community assembly)

Evolutionary relationships - importance of coevolved human symbionts (the long-term adaptation and interdependency between humans and specific microbial species)

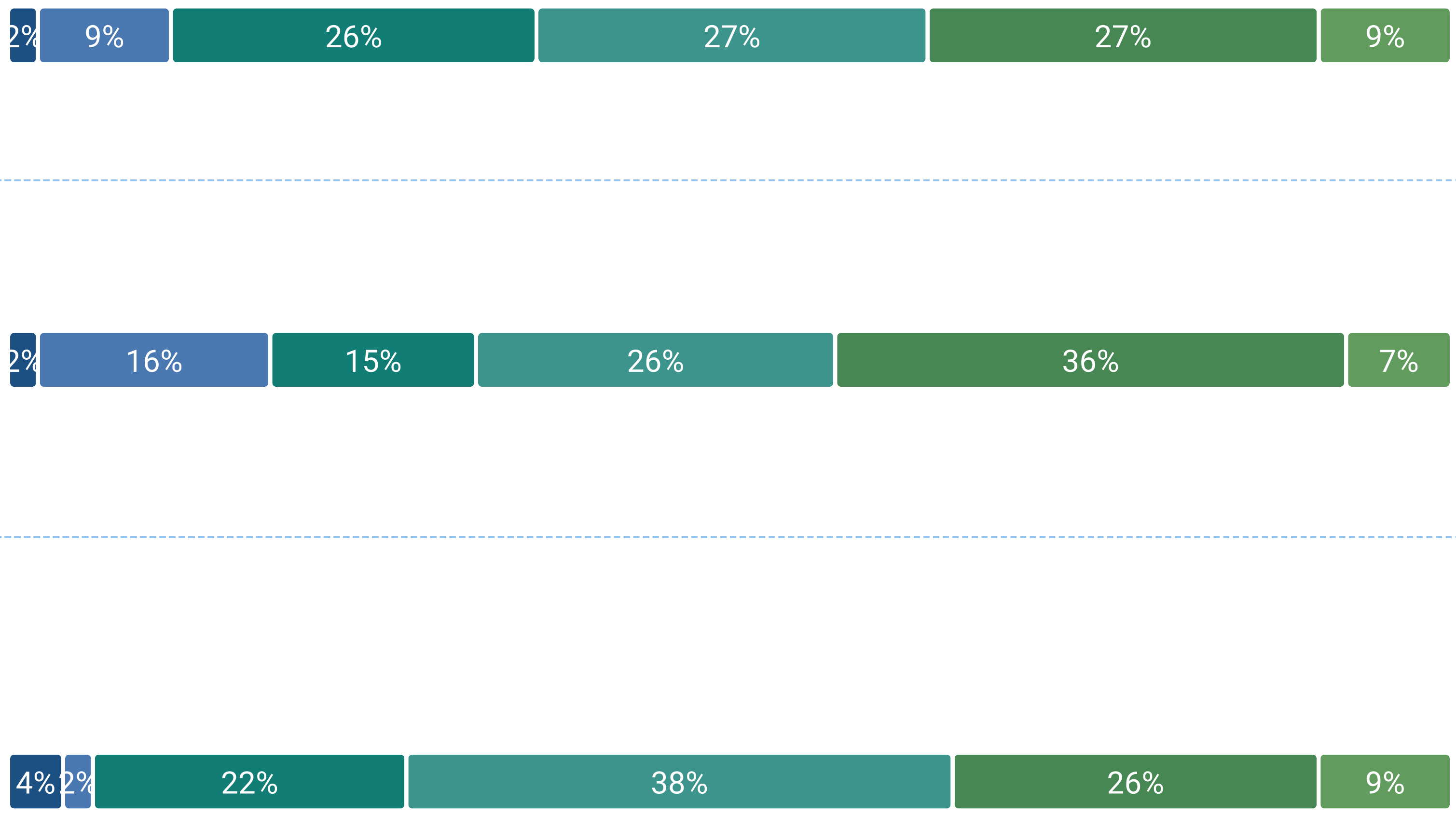

Rows	1	2	3	4	5	I don’t know
Stochasticity of the process (randomness in initial colonization and subsequent changes)	1	5	14	15	15	5
Priority effects - order of arrival (the impact of early colonizers on subsequent microbial community assembly)	1	9	8	14	20	4
Evolutionary relationships - importance of coevolved human symbionts (the						

human symbionts (the long-term adaptation and interdependency between humans and specific microbial species)	2	1	12	21	14	5

If you have comments or suggestions, please let us know!

Number of submissions: **3**

Submissions

In general, our understanding of the ecological and evolutionary principles at play in the colonization of the neonatal/infant gut is still poor.

Always more ecological studies are awesome

This is a highly contentious topic, as the success of colonisation depends on the extent to which the environment can provide strains that can colonise the gut and what microbial features determine this. Moreover, it also involves understanding the recipient factors that define more or less resistance to colonisation. They can be associated with host genetics and host habits and diets, but also with the current community. The level of complexity of this kind of questions make this a really interesting topic.

VIII. Maternal Factors

Which maternal factors relevant to the early life microbiome should be better studied?

Rows

maternal medications (antibiotics)

maternal medications (non-antimicrobial drugs)

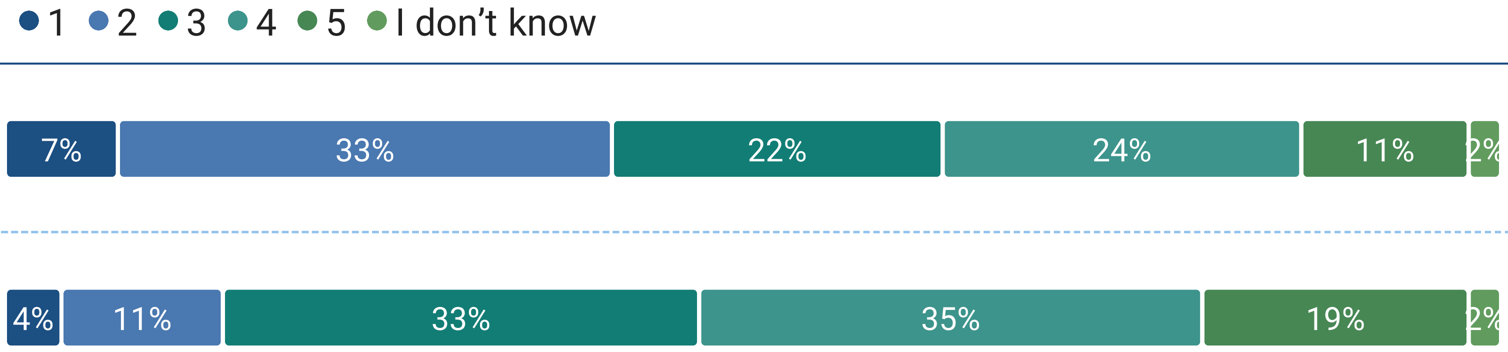

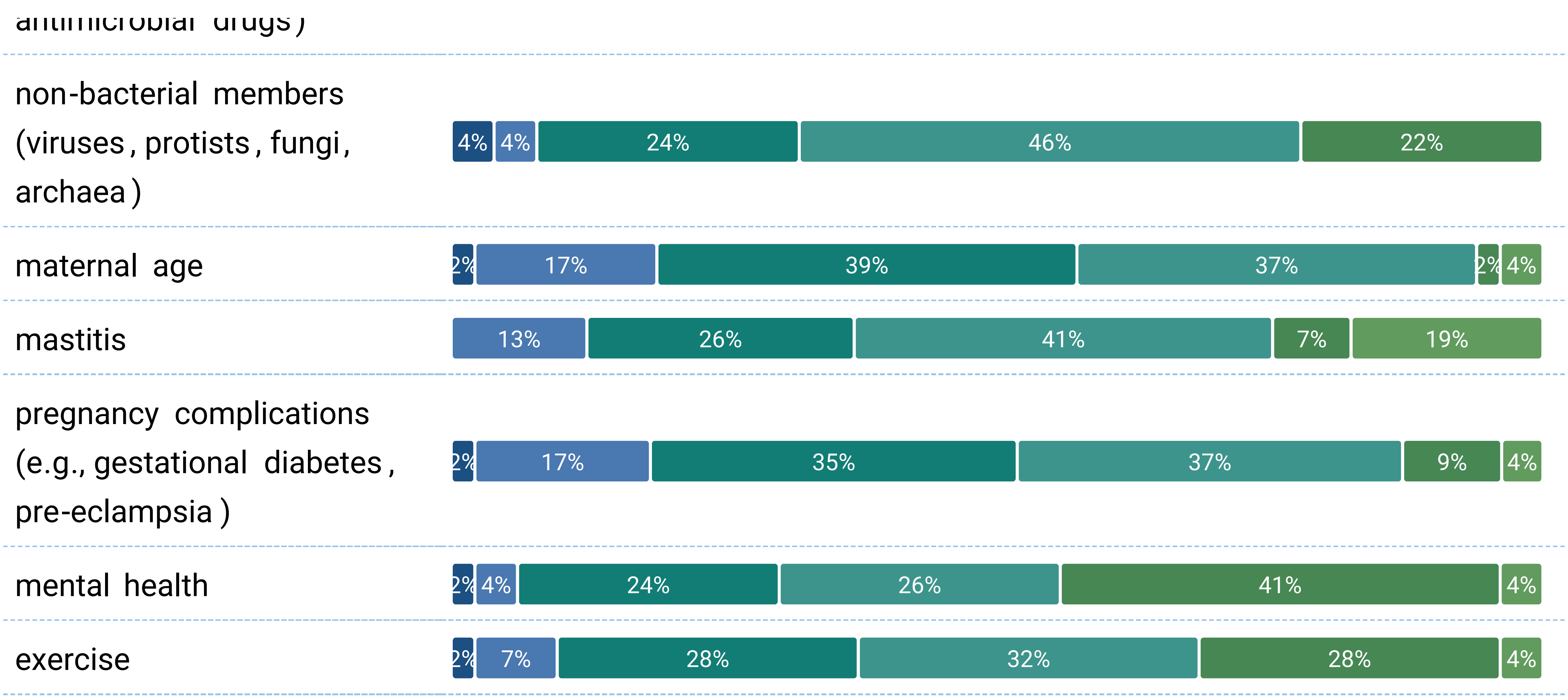

Rows	• 1	• 2	• 3	• 4	• 5	• I don't know
maternal medications (antibiotics)	4	18	12	13	6	1
maternal medications (non-antimicrobial drugs)	2	6	18	19	10	1
non-bacterial members (viruses, protists, fungi, archaea)	2	2	13	25	12	0
maternal age	1	9	21	20	1	2
mastitis	0	7	14	22	4	10
pregnancy complications (e.g., gestational diabetes, pre-eclampsia)	1	9	19	20	5	2
mental health	1	2	13	14	22	2
exercise	1	4	15	17	15	2

If you have comments or suggestions, please let us know!

Number of submissions: **2**

Submissions

For mastitis, I believe there are studies, but the sample sizes are usually small because of the definitions used for mastitis. Similarly the sample sizes for pregnancy complications have also been small.

Diet, supplements, pre- and probiotics, length of those changes before and during pregnancy

And all of the above for the father and their sperm

IX. Body sites

What body sites should be sampled in future cohorts?

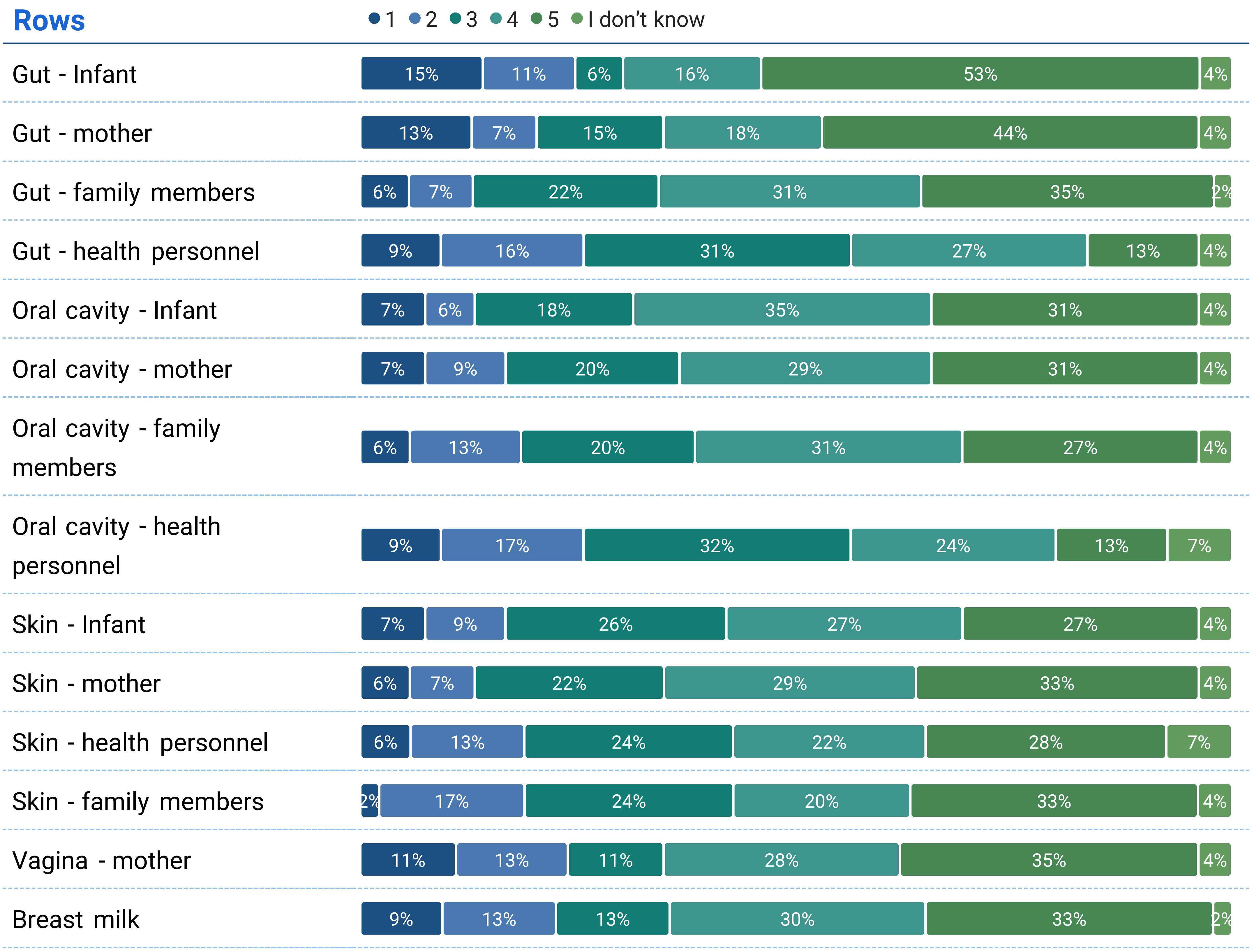

Rows	1	2	3	4	5	I don’t know
Gut - Infant	8	6	3	9	29	2

Gut - mother	7	4	8	10	24	2
Gut - family members	3	4	12	17	19	1
Gut - health personnel	5	9	17	15	7	2
Oral cavity - Infant	4	3	10	19	17	2
Oral cavity - mother	4	5	11	16	17	2
Oral cavity - family members	3	7	11	17	15	2
Oral cavity - health personnel	5	9	17	13	7	4
Skin - Infant	4	5	14	15	15	2
Skin - mother	3	4	12	16	18	2
Skin - health personnel	3	7	13	12	15	4
Skin - family members	1	9	13	11	18	2
Vagina - mother	6	7	6	15	19	2
Breast milk	5	7	7	16	18	1

If you have comments or suggestions, please let us know!

Number of submissions: **2**

Submissions

While the maternal pregnancy gut microbiome has been relatively well sampled, I would argue that most birth cohorts tend to omit the postpartum gut microbiome.

It would be beneficial to explore the link between breast milk metabolomic profiles and the succession dynamics in the baby's gut, verifying whether components in the milk tend to be similar to components in the mother's and infant's colonic mucus layer (I would even focus on the mucus within the crypts).

Of course, the comparison with the metabolic profile of formulas makes it even more interesting.

X. Summary

Which factors should be prioritised to study in future

Which factors should be prioritised to study in future microbiome studies?

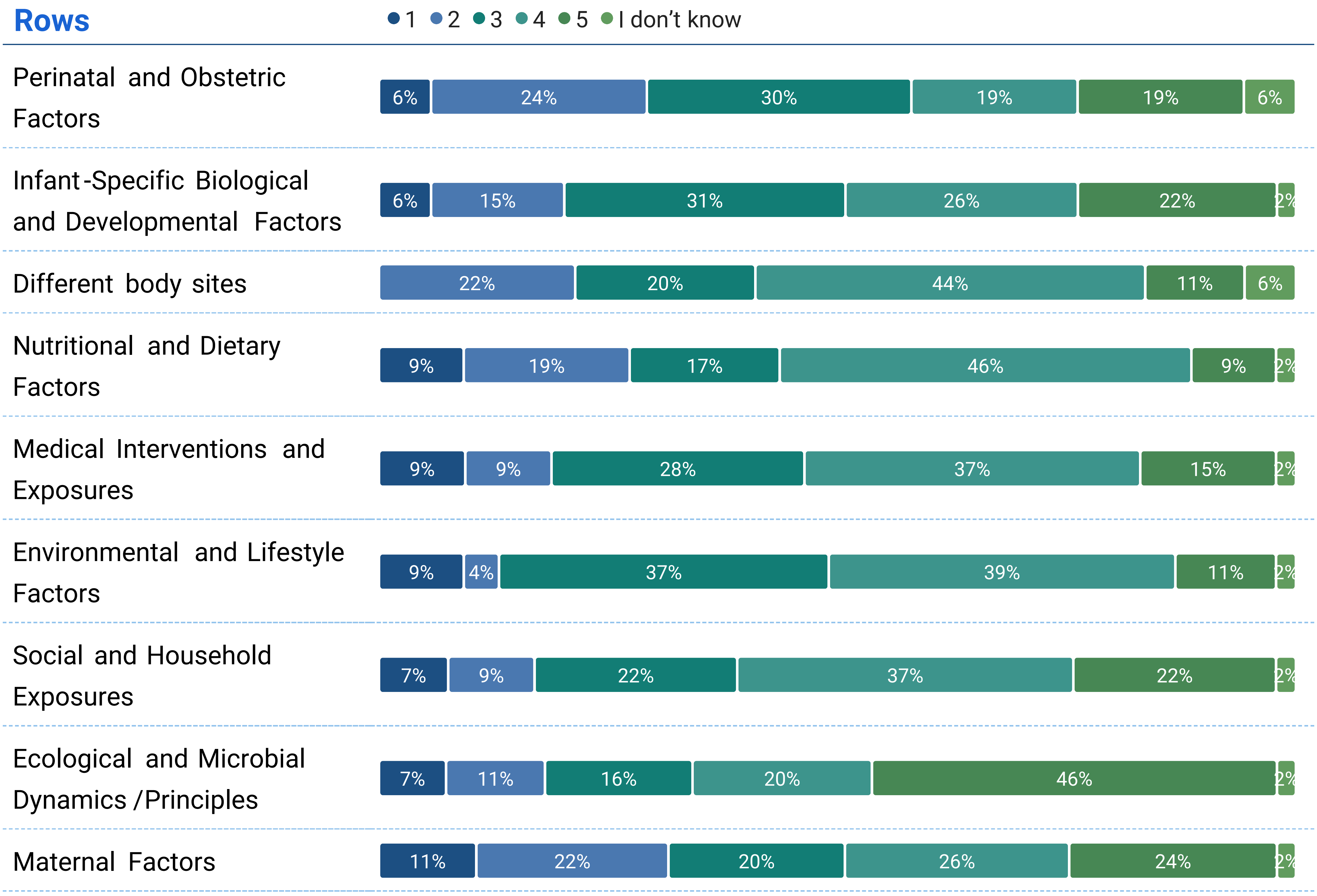

Rows	1	2	3	4	5	I don't know
Perinatal and Obstetric Factors	3	13	16	10	10	3
Infant-Specific Biological and Developmental Factors	3	8	17	14	12	1
Different body sites	0	12	11	24	6	3
Nutritional and Dietary Factors	5	10	9	25	5	1
Medical Interventions and Exposures	5	5	15	20	8	1
Environmental and Lifestyle Factors	5	2	20	21	6	1
Social and Household Exposures	4	5	12	20	12	1

Ecological and Microbial Dynamics /Principles	4	6	9	11	25	1
Maternal Factors	6	12	11	14	13	1

If you have comments or suggestions, please let us know!

Number of submissions: **3**

Submissions

Many of these topics are highly relevant and deserve further study - but could be and in some cases should be different studies/study designs. E.g. ecological and microbial dynamics principles would need more dense sampling than some of the other topics.

What composes maternal factors is unclear to me. Cannot say how well that has been studied, as some factors have been and some perhaps have not.

One question that comes to my mind is what mechanisms are behind maternal effects. To what extent is this about similar genetics, shared environments or breastfeeding? What are the functions enriched in bacteria with higher maternal effects, and how does it elucidate the main mechanisms behind it? Additionally, to what extent do components in the milk that are more or less similar to the ones in the colonic mucus act as a bridge or not between the genetic influence behind maternal effects?

A bit of your background..

What is your experience in the microbiome field?

Rows	Select if fitting
An undergraduate student	100%
A graduate student (MSc or PhD)	100%
Postdoctoral fellow	100%
Assistant Professor	100%
Associate Professor	100%
Professor	100%
Researcher, Senior Researcher or Staff scientist in research institutes	100%
Researcher, Senior Researcher or Staff scientist in higher education	100%
Researcher, Senior Researcher or Staff scientist in Industry	
Other	100%

Rows	Select if fitting

An undergraduate student	1
A graduate student (MSc or PhD)	21
Postdoctoral fellow	11
Assistant Professor	6
Associate Professor	7
Professor	7
Researcher, Senior Researcher or Staff scientist in research institutes	4
Researcher, Senior Researcher or Staff scientist in higher education	1
Researcher, Senior Researcher or Staff scientist in Industry	0
Other	2

If you choose "other" please specify.

Number of submissions: **2**

Submissions

Medical microbiologist
Independent professional with microbiology & bioinformatics background active for 5+ years in microbiome startups/scaleups

Please let us know the institution and country of your main employment.

Number of submissions: **39**

Submissions

Quadram Institute, UK
University of Helsinki Finland
UZA/UA, Belgium

[illegible]

University of Turku, Finland

Bar-Ilan university, Israel

University of Calgary

UMCG Gronigen

University of Amsterdam, Netherlands

University of Valencia, Spain

MUMC+ - Netherlands

UCSF - USA

UiT, Norway

Uppsala University, Sweden

Karolinska Institutet, Sweden

Ireland

University of Oslo, Norway

Uppsala University, Sweden

Technical University of Denmark, Denmark

Karolinska Institutet/University of Antwerp

UPF, Spain

University medical center, Groningen

Pompeu Fabra University, Spain

University of Helsinki, Finland

Helsinki University, Finland.

University of Helsinki, Finland

University of Groningen, the Netherlands

Chalmers University of Technology, Sweden

Universitat Pompeu Fabra, Spain

Finland

University Medical Center Groningen, the Netherlands

UMCG, the Netherlands

QIB, Norwich

Centre for Genomic Regulation (Spain)

UiT, Norway

University of Antwerp (prior knowledge from University College Cork)
Maastricht university , NL
Belgium
Quadram Institute Bioscience, UK
Umeå University, Sweden

What is your connection to the early life microbiome (maternal microbiome during pregnancy and the infant microbiome)?

Number of submissions: **55**

Submissions	Count	% of submissions	Chart
Primary research interest	41	74.5%	74.5%
Somehow overlaping with my main research field	14	25.5%	25.5%
Curiosity	4	7.3%	7.3%
Have heard about it but don't know much on the topic	1	1.8%	1.8%
Other	0	0%	0%

If you choose "other" please specify.

Number of submissions: **1**

Submissions
Currently, my main client develops HMOs

Thank you for making it all the way to the end and for your contributions!
